# The perspectives of broadband metasurfaces and photo-electric tweezer applications

**DOI:** 10.1515/nanoph-2021-0711

**Published:** 2022-01-24

**Authors:** Geon Lee, Eui-Sang Yu, Yong-Sang Ryu, Minah Seo

**Affiliations:** Sensor System Research Center, Korea Institute of Science and Technology (KIST), Seoul 02792, Republic of Korea; Department of Physics and Astronomy, Seoul National University, Seoul 08826, Republic of Korea; Brain Science Institute, Korea Institute of Science and Technology (KIST), Seoul 02792, Republic of Korea; KU-KIST Graduate School of Converging Science and Technology, Korea University, Seoul 02841, Republic of Korea

**Keywords:** biosensor, dielectrophoresis, metasurface, optical sensing, plasmonic trapping, tweezer

## Abstract

With strong demands of real-time monitoring of biomolecules or environmental pollutants, overcoming technical hurdles on control and detection of freely diffusive nanoscale objects become a question of issue to solve in a variety of research fields. Most existing optical techniques inevitably require labeling to the target material, which sometimes denature the measuring biomaterials. For highly efficient real-time monitoring without complicated pretreatment or labeling, many successes in development of label-free or non-destructive detection techniques via increased sensitivity were accomplished by the additional structures. Metasurface-based two-dimensional photonic/electric devices have recently represented extraordinary performances in both manipulation and sensing for various small particles and biochemical species, repeatedly overcoming the limit of detection achieved right before. In parallel, various metasurface-based devices were also introduced promoting transportation of targets into optical hotspot sites, overcoming diffusion limits. We noted this point, therefore, reviewed two major research fields such as metasurface-assisted material sensing and transportation technologies that have contributed to present prospective sensing technologies, then showed perspective views on how great synergy can be created when two technologies are cleverly integrated. Recently, a trend of conceptual merging of optical detection and transporting schemes beyond both diffraction limit and diffusion limit leads to a creation of exceptional performance in molecular detections. In this review, the trends of the latest technologies accomplishing this purpose by hybridization of various composite materials and functional metasurfaces will be introduced.

## 1 Introduction

The metasurface consisting of an arrangement of artificial ‘unit atoms’ are generally designed with metals or dielectric materials especially within a size much smaller than the wavelength of the broadband electromagnetic wave. The meaning of the term ‘meta’ can be interpreted as altered or beyond with respect to its design conjugated by more than two different materials. This periodic array of unit atoms may result in completely new optical/electrical properties (dielectric constants) that does not exist in nature at specific wavelengths. The shape, structure, size, direction, and alignment of this unit atom can therefore affect various optical phenomena, including scattering, interference, traveling direction, and diffraction. Metasurface research can be considered as an excellent multidisciplinary and convergence science such as electromagnetics, optics, solid physics, microwave engineering, optoelectronics, material engineering, and nanoscience. In particular, the metasurface fabrication was realized at millimeter and micrometer regimes for the first time, since it is relatively easy to handle and manufacture such sized structures in an early stage. With the assistance of the remarkable development of nano-fabrication technology to date, the operation wavelength has been getting shorter, thus covering most of the wavelength regime.

This interesting concept, which began with theoretical prediction, has been experimentally realized recently and has been used for various purposes, gradually expanding practical applications. As one of the promising applications, electromagnetic field engineering in a wide wavelength band using various types of metasurfaces is suitable for effectively detecting trace amounts of small material. Last few decades, a major trend of molecular analyzers over the metasurface has been led for designing unique photonic nanostructures together with particle adhesion nearby the photonics hotspots. While a branch of photonic researchers tries to improve molecular detectability through novel nanostructures with extraordinary photonic performance such as sensitivity, other mainstream research trends were led by molecular captures and concentration in the intended area for enhancement of detectability. Based on physicochemical understanding of surface science, photonics scientists collaborate with multidisciplinary researchers to pinpoint the target molecules in the vicinity of the optical hotspot. However, a majority of research works heavily rely on evaporation-driven particle concentration and trapping. Considering that most biomolecules are viable under liquid conditions, a new strategy for molecular detection underwater is highly required for leading the future bio-photonic research field. Recently, a few approaches that combine particle captures with optical/electrical detection were intensively explored using plasmonic trapping of nanoscale objects by exposing incident light at the sensing spots such as nanoantenna or nanodot. This opened a route for simultaneous molecular detection together with particle control. However, a restricted regime of laser spotting area results in low throughput of molecular capability, which is disadvantageous for spreading their unique techniques toward wide ranges of conventional particle concentration tools.

Here, we introduce various molecular detection schemes employed by metasurfaces and resonant nanostructures at the broadly defined wavelength regime, as they conserve the inherent molecular characteristics with improved sensitivity. Such sensing techniques can be combined with targeted particle manipulation techniques that have been actively studied recently. Various optical sensor technologies based on the metasurfaces that analyze the optical characteristics of trace molecules are introduced. Finally, next-generation sensing technology allows us to precisely control the dynamic movement of particles and measure them at the same time, by overlapping the signal hotspot, even controlling the behavior of small particles under the water environment are reviewed as well.

## 2 Metasurface enhanced molecular sensing

**Figure 1:**
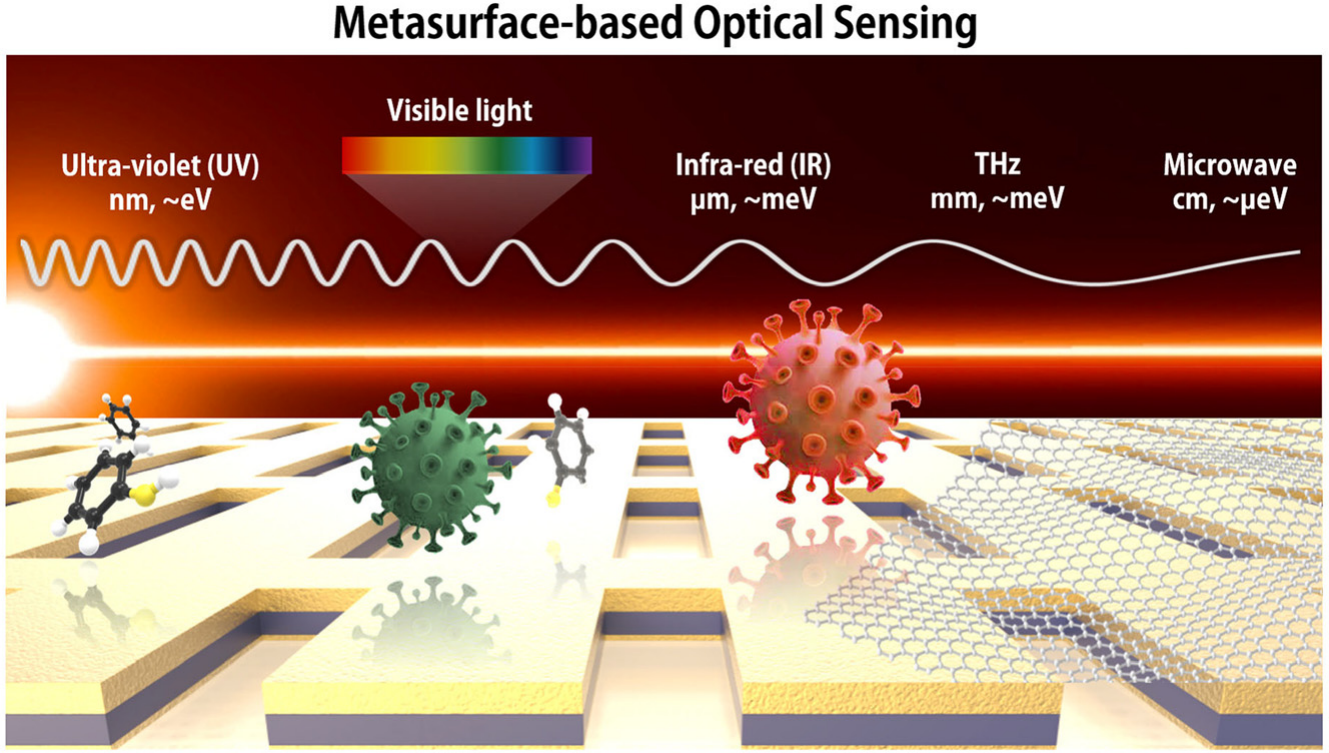
Conceptual illustration of biochemical substance monitoring over the metasurface-based structures at the ultrabroad band of electromagnetic waves. Significantly improved light-molecule interactions by the metasurfaces and increased absorption cross-sections of the molecules provide improved sensing performances even in extremely low density of the molecules as described.

The fundamentals of monitoring the trace of biochemical molecules and their assemblies using the metasurface-based nanostructures (consisting of all-dielectric, metallic plasmonic, and hybridized metasurfaces with atomically thin layers) were illustrated in Figure 1. The metasurface-induced extraordinary enhancement of optical signal (either transmission or reflection) allows detection of extremely small molecules by offering significantly improved light-molecule interactions and absorption cross-sections of the molecules. Present optical sensors with various transduction schemes including evanescent wave engineering, surface plasmon resonance behavior, or immobilization strategies can overcome limitations in analyzing trace amounts of biologically or environmentally related compounds of small molecular weight in extremely low density. While maintaining the label-free and non-contact schemes, which is the most attractive part of optical sensors, much improved sensing performances are represented with the assistance of metasurfaces as additional signal enhancers.

In this chapter, various detection applications for higher molecular sensitivity employed by metasurfaces and resonant nanostructures at the broadly defined wavelength regime are introduced without consideration of molecular damage or denature. The fundamentals of the metasurface-based optical sensing techniques in terms of metasurface geometry, composition materials, and the fabrication methods are intensively studied.

### 2.1 Metasurfaces fundamentals and their resonance behavior

Metasurface-based plasmonic structures which have sharp resonance behavior at ultrabroad band regime have been widely explored and proven to be one of the most successful applications of sensing to date. Such metasurface includes hybrid structure with various material combinations including metals, semiconductors, and nonlinear optical crystals. In particular, the geometrical patterns of the metasurface structures down to nanometer levels (e.g., subwavelength resonators, nanowires, nanoparticles, and nanoantennas) overwhelmingly contributed to the improvement of the optical functionality. This is attributed to the increased surface-to-volume ratio and electromagnetic field localization effects in the extreme near field. Metasurfaces can be classified into two main categories depending on how a unit artificial atom is designed. First, unit artificial atoms and their properties determine resonance behavior of electromagnetic properties at a certain wavelength (resonant frequency). Second, a broadband metasurface can be produced by adjusting the electrical and magnetic boundary conditions in the geometric shape of the unit artificial atom. A majority of metasurface studies have been conducted within the former case focusing on analyzing the resonance characteristics. However, desired characteristic values are analyzed at the limited and a narrow wavelength band, as it intrinsically loses significant optical signals from the other bands. On the other hand, broadband metasurfaces (i.e., later case) is highly advantageous over a wide wavelength showing desired optical performance and characteristic values. However, there is still a disadvantage from a large frequency dispersion. It is, therefore, important to design metasurfaces appropriately according to the desired application.

**Figure 2:**
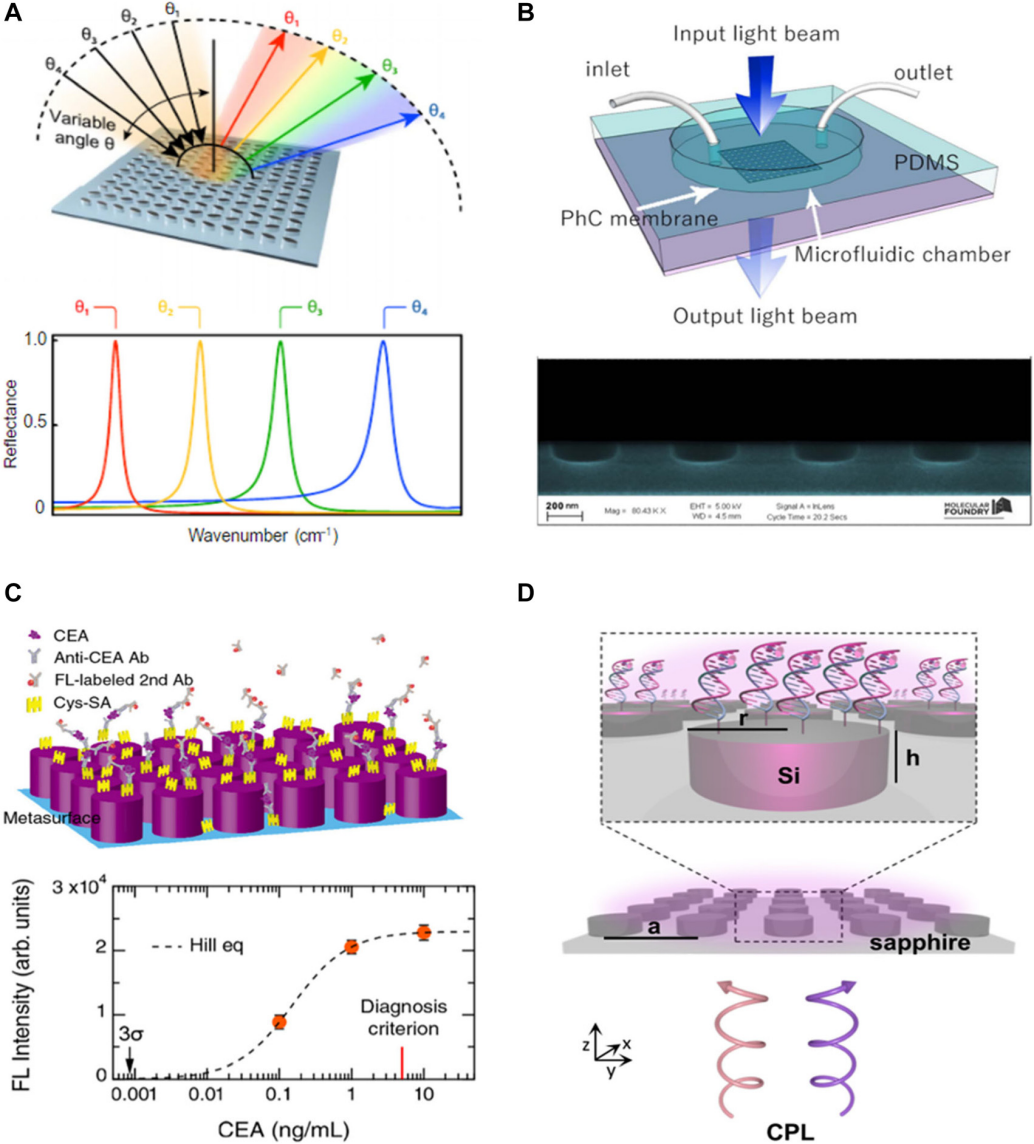
All-dielectric metasurface enhanced sensing. (A) A broadband on-demand resonance behavior for every incidence angle (bottom) from high-*Q* germanium-based metasurfaces (top). Reproduced with permission from ref. [1], copyright 2019, AAAS (American Association for the Advancement of Science). (B) Polydimethylsiloxane (PDMS) microfluidic chamber bonded to the PhCM (top). The air cylindrical holes arranged in a silicon nitride film (bottom). Adapted with permission from ref. [2], copyright 2018, Optical Society of America. (C) CEA molecule detection by dielectric metasurface (top). FL-intensity plot versus CEA concentration (orange closed circle) with fitted curve (dashed curve) using the Hill equation (bottom). Adapted with permission from ref. [3], copyright 2020, American Chemical Society. (D) Chiral molecule detection using dye/DNA functionalization with periodic silicon disk-arrays. Reproduced with permission from ref. [4], copyright 2020, American Chemical Society.

Meanwhile, metallic plasmonic structures lead a trend of metasurface studies at an early stage, all-dielectric material engineering with semiconductors or nonlinear optical crystals in recent days has revolutionized the perception of metasurfaces for sensing applications. As one of the examples, an angle-scanning technique via dielectric materials was applied to detect molecular substances (Figure 2A). By tuning the incidence angle of light, the resonance frequency can be effectively tuned, as it allows broadband spectral response. The resonance frequency was determined to fit the vibrational mode of target analytes, with a high *Q*-factor of 200 and obtained spectral resolution about 1.5 cm^−1^ The dielectric metasurface with DNA aptamer, the target sample, odontogenic ameloblast-associated (ODAM) protein, which is a unique element of calcifying epithelial odontogenic tumors (CEOTs) was investigated with sensitivity of 0.27 pg/mm^2^ and limit of detection (LOD) of 3000 molecules/mm^2^ [1]. Using dielectric materials with bound states in the continuum (BIC) resonance, a very high *Q*-factor, 2,000, was achieved producing figure-of-merit (FOM) up to 445 (Figure 2B). There is a merit of using dielectric materials for the metasurfaces, free from the strong energy loss, which is unavoidable in the metallic plasmonic structure. Thus, the ultra-low-molecular-weight of biphenylthiol (BPT) self-assembled monolayers (less than 1 nm in thickness with 1 μM in concentration) could be detectable [2].

For better biosensing performance, another type of metasurface, all-dielectric metasurface conjugated with a fluorescence platform was suggested for detection of immunoglobulin G (IgG), p53 antibodies and carcinoembryonic (CEA) cancer marker at very low concentrations in order of 10 fM (Figure 2C). It presents stable responses even for actual liquid-biopsy including human serum. Regarding the real medical diagnosis criterion is 5 mg/ml, the obtained LOD, 0.85 pg/ml is quite promising to the practical uses [3]. Geometrical factors like chirality are also important parameters to be carefully considered, since many biomolecules including protein, amino acids and carbohydrates have non-mirror symmetry in nature. Nevertheless, it is still hindered to measure the chiral property directly, because the chirality interaction of molecules and light is too weak. Fortunately, such faint signals can be enhanced by metasurfaces composed with periodic arrays of silicon disks, triggering electric and magnetic dipolar Mie resonances (Figure 2D). The fluorescence labeling of oligonucleotide strands to the silicon disk additionally enhanced molecular circular dichroism (CD), depending on the DNA conformational change. Opposite signs for double-stranded DNA (dsDNA) and single-stranded DNA (ssDNA) were obtained as expected [4].

The most popular scheme is using metals to produce plasmonic effects based on surface plasma wave (SPW) propagating by following: $\beta =k_{0}\sqrt{\epsilon_{m}\epsilon_{s}/\left(\epsilon_{m}+\epsilon_{s}\right)}$ with propagation coefficient β , angular wavenumber in free space *k*_0_ and dielectric constant of metal ( $\epsilon_{m}$ ) and substrate ( $\epsilon_{s}$ ) [9]. Especially, surface plasmon resonance (SPR) from the sub-wavelength metal structure has emerged as a promising method for various applications such as extraordinary optical transmission (EOT), photocatalysis, solar cell and biomedical sensing. As mentioned earlier, the studies on metasurfaces first began in millimeters and microwaves in the early 2000s. This was due to technical limitations such as how to produce metasurfaces in sub-wavelength scale, or lack of reliable measurement methods. However, the current status of nanofabrication and highly developed optical measurement technologies enable metasurfaces to operate in wider frequency regimes. The structure and operating wavelength range are from tens to hundreds of nanometers, finally. Metasurface structures, which started to be developed with the split ring resonator (SRR) earlier, gradually have been varied in shape including a modified or overlapped ring structure rather than a simple SRR structure [10], a repeated pattern of the ring structure, a chiral patterned structure [11], hyperbolic dispersion structure, or a straight antenna-shaped structure. This may include a metal (positively embossed), or conversely, a metal in the periphery with a filled dielectric in antenna shape. Also, optical resonance characteristics in the transmission/reflection of incident light were studied theoretically such as SRR $f_{SRR}=1/2\pi \sqrt{c_{1}a^{2}+c_{2}}$ [12] and straight antenna $f_{antanna}=c/2\pi l\sqrt{2\left(n_{sub}^{2}+n_{medium}^{2}\right)}$ [13] where *a* and *l* denote dimension length of structure while *n* is refractive index, *c* is light-speed and *c*_1_ and c_2_ is length independent parameter from LC model. Thus, the working frequency can be tuned freely according to the purpose. Furthermore, the metasurfaces had a simple combination of metal and dielectric substrates at the beginning, and gradually their composition varied into a combination of materials having various dielectric constants such as semiconductors, phase shift materials, or absorbers. It has been theoretically and experimentally explored that such metal-based metasurfaces with a combination of various materials exhibit their unique performance optically and electromagnetically.

**Figure 3:**
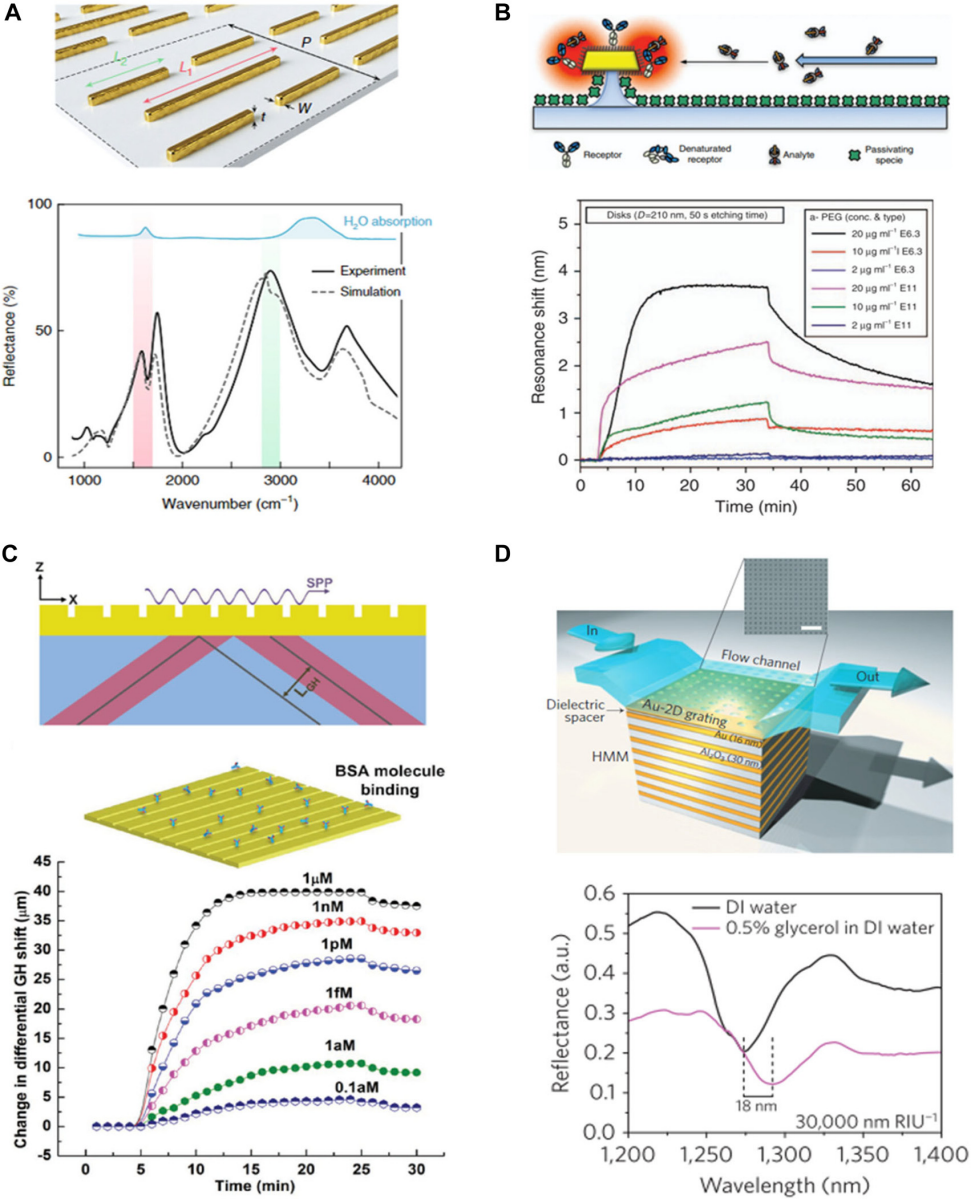
Plasmonic metasurface for high sensitive molecule sensing. (A) Multi-resonant mid-IR metasurface (top) and experimental reflectance spectra (bottom). Reproduced with permission from ref. [5], copyright 2018, Springer Nature. (B) Pillar-supported disk structure increases the effective sensing volume and performance (top). Real-time sensing of interaction of two types of IgGs as antibody concentration (bottom). Adapted with permission from ref. [6], copyright 2017, Springer Nature. (C) The diagram of nanogroove hyperbolic metasurface (top) and plasmonic sensing of binding interaction of bovine serum albumin molecule by the change in differential GH shift (bottom). Reproduced with permission from ref. [7], copyright 2017, John Wiley and Sons. (D) A graphical representation of the GC-HMM sensor device with a fluid flow channel (top). Measured time-varying wavelength shifts while 10 pM biotin injection (bottom). Adapted with permission from ref. [8], copyright 2016, Springer Nature.

Developed plasmonic metasurface, in particular operating at long-wavelength regime, have been used label-free bio-molecule sensing, issued near 10 years due to its non-destructive and non-ionized nature (relatively low photon-energy, ∼meV). The typical absorption spectra of vibrational modes under various molecules exist at these broadband wavelengths, thus vibrational spectroscopies are commonly established for assessing molecular motion and fingerprinting species. Altug et al. designed multi-resonance based metasurfaces for chemically specific sensing (Figure 3A) [5]. The reported infrared (IR) sensor consists of two different lengths of metallic nanoantenna-arrays which provide up to 1000-fold near-field enhancement over a broad IR spectrum. Each length of nanoantenna is conducted to target molecule-specific frequency which is determined by vibrational modes of both the amide I, II, and the CH_2_, CH_3_ absorption bands. Using this unique platform, real-time absorption change could be monitored during lipid membrane formation through CH_2_ group of streptavidin, allowing to analyze the disruption of peptide-induced membrane and releasing of neurotransmitter cargo from synaptic vesicle mimics. The key conceptual idea for sensing purposes using metallic structure has grown based on the principle of localized surface plasmon resonance (LSPR) activated by the delicate variation in the extinction spectra due to the refractive index change of the metallic surrounding medium [14], [15], [16]. Targeted molecule samples can be coated onto a metal structure in the form of a thin film, and the amount of molecule can be counted by the change of the LSPR signal. In that experimental scheme, however, unintended molecule adsorption to the dielectric substrate, it is difficult to avoid depletion of the target molecules and disturbance of LSPR induced evanescent electromagnetic field. To overcome this limit, Käll et al. suggested disk-shaped gold nanoparticles on nanopillars which are decoupled from the substrate resulting in nonspecific adsorption (Figure 3B) [6]. Nanopillar-supported gold nanodisks and additional neutravidin (NTV) coating to prevent depletion of adhered biomolecule to substrate, could make even 2 μg/ml of antibody (E11 and E6.3 IgGs) observable. The metallic groove nanostructures which is one of hyperbolic metamaterials (HMMs) is a new type of 1D nanowire-arrays which is able to confine the surface plasmon waves to the metal surface and thus, potential sensing platform [17]. HMM induced epsilon-near-pole (ENP) resonance can detect low weight biomolecules via huge phase change by even a tiny perturbation of the surroundings. Goos–Hänchen (GH) shift (higher order derivation of phase) of the TM-polarized light is a good example, resulting in sensitive detection of diluted bovine serum albumin (BSA) solution as low as 0.1 aM (Figure 3C) [7]. Another HMM structure fabricated by stacking 16 alternating gold/Al_2_O_3_ multilayers represents four high Q-factor reflectance minima by highly confined bulk plasmon polaritons in hyperbolic region (*λ* > 520 nm) and SPPs in elliptical region (*λ* < 500 nm). This coupling condition varies by surrounding medium causing resonant wavelength shift, 30,000 nm/RIU at near IR. Such a high FOM enables the detection of biotin (244 DA) even for 10 pM concentrations (Figure 3D) [8].

### 2.2 Metasurface applications and their performances

One of the most common optical applications of metasurface structures is detection of target materials. Constant values of optical signals become changed in response to the molecular exposure followed by their surface attachment, resulting in additional shifts either of intensity or resonant wavelength (=frequency). Taking advantage of the working principle, trace amounts of molecules can be monitored in a non-contact and non-labeled manner. Where information of various complex refractive index values for substances is gathered in a wide wavelength region, quantitative and qualitative analysis of substances is possible. In this chapter, the sensing applications that operate with different mechanisms depending on the optical characteristics of each wavelength regime will be introduced.

**Figure 4:**
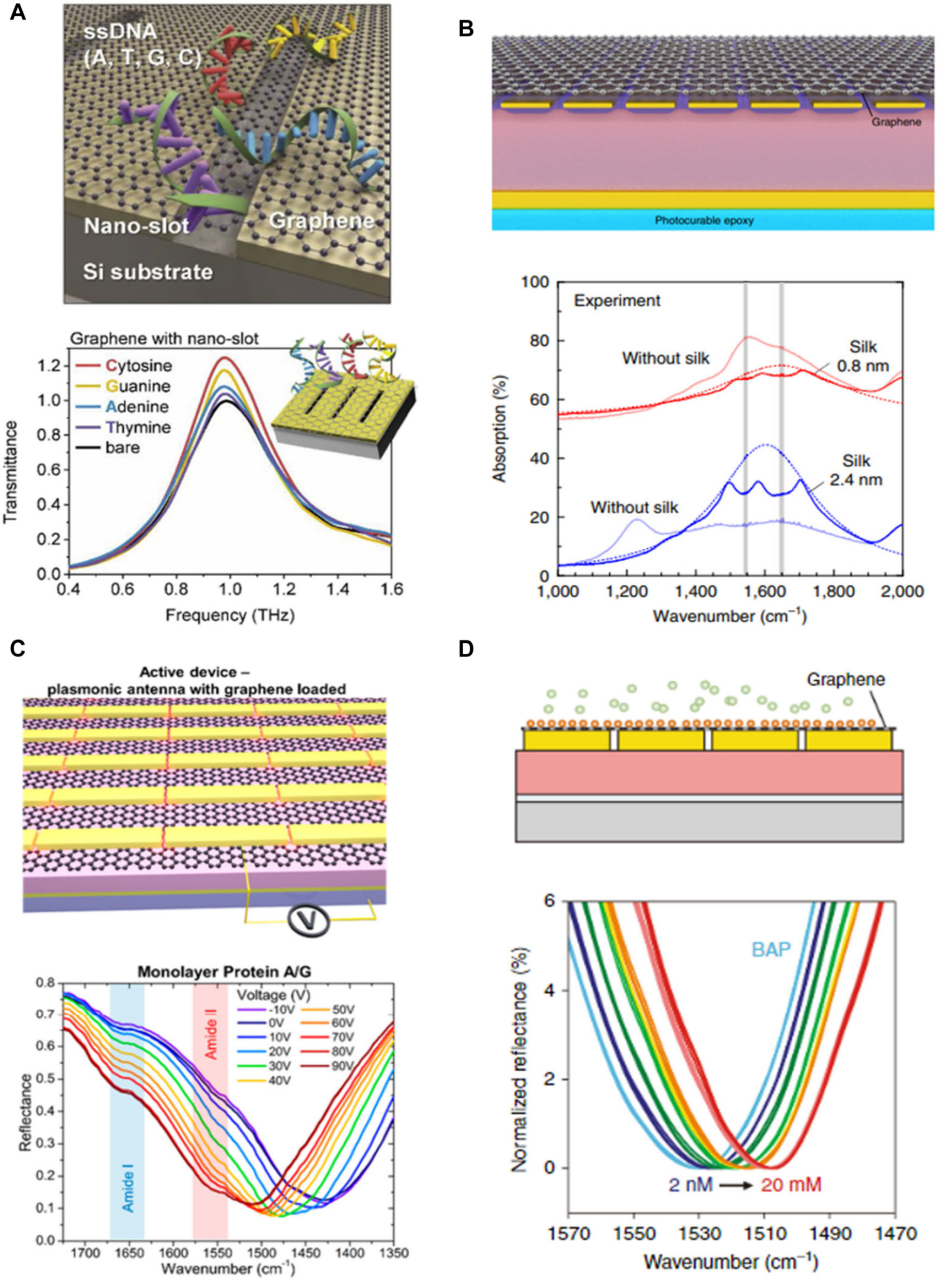
2D material combined metasurface sensor. (A) DNA adsorption on the graphene-combined nanoslot metasurface (top). Change in THz transmission spectra due to ssDNA on a graphene covered nanoslot metasurface (bottom). Adapted with permission from Ref. [18], copyright 2020, Elsevier. (B) Illustration of acoustic plasmon resonator architecture (top) and measured absorption spectra about resonators with and without a silk film (bottom). Lorentzian fitting curves are shown as well (dashed lines). Adapted with permission from Ref. [19], copyright 2019, Springer Nature. (C) Active metasurface sensors for protein fingerprinting (top) and measured reflectance spectra of monolayer protein A/G and controlled Fermi level of graphene via a gate voltage (bottom). Reproduced with permission from Ref. [20], copyright 2019, American Chemical Society. (D) Graphene-metallic hybrid metasurface sensor (top) and spectral responses of various concentrations of glucose on the hybrid metasurface (bottom). Reproduced with permission from Ref. [21], copyright 2018, Springer Nature.

Monolayers of two-dimensional films including graphene plasmons (GPs), especially, which enables deep-subwavelength electromagnetic confinement, have been intensively exploited for various applications including metasurfaces for sensors [22]. Recently, the graphene hybridized nanoslot demonstrated remarkable change of transmission in accordance with four ssDNAs, related to the different binding energy and adsorption characteristics (Figure 4A). Considering the quantity of soluted ssDNA and adsorbed region, it can be inferred to 38.2 fM of ssDNA detectable. Electrostatic depolarization, which is induced by biomolecule adsorption on the graphene, modificates the Fermi level or conductivity of graphene, so that optical transmittance is effectively changed [18]. Acoustic graphene plasmons (acoustic GPs) are also observed when graphene is placed on a metal surface (Figure 4B). Using acoustic GP resonators, dramatically increased plasmon resonances captured up to 94% of absorption in mid-IR, enabling ultrasensitive detection of few layer protein films. By adapting acoustic GP resonators, the absorption of vibrational mode is increased as 13.2% for amide I band and 11.6% II band with high signal-to-noise ratio [19].

The active control of graphene plasmon resonance is also one of the important issues in using monolayers of films, suggesting broadband tuning for molecular fingerprinting [23]. The gold nanorod-array on monolayer graphene devices demonstrated active tuning by adjusting bias voltage which accurately overlaps the metasurface resonance with biomolecules’ vibrational modes (Figure 4C). Using this device, amide vibration mode of protein IgG can be obviously resolved and tiny quantities of IgG as 30 pM can be efficiently detected despite very weak fingerprints. The results are validated as compared with conventional attenuated total reflection (ATR)/Fourier transform infrared spectroscopy (FTIR) measurements showing enhanced sensitivity more than 4 orders of magnitude [20]. Such hybrid metasurface-based sensors consisting of monolayer graphene and metallic nanoantenna-arrays can contribute to improving the sensitivity of typical SPR optical sensors as well. Gold nanorod type of dipole antenna-array covered by monolayer graphene revealed strong resonance behavior at mid-IR wavelength. In recent work from Lin et al., boronic acid-pyrene (BAP) with an empty orbital, was used as an electron-withdrawing, initially p-doped graphene (Figure 4D). After such treatment, injected glucose weakens electron-withdrawing capability through direct binding with BAP and thus leading to red-shift of resonance. Distinctly resolvable red-shift of ∼1.5 cm^−1^ was measured from 2 nM (0.36 ng/mL) glucose exposure and LOD was improved by almost five orders of magnitude as compared to existing binding-based glucose sensors [21].

**Figure 5:**
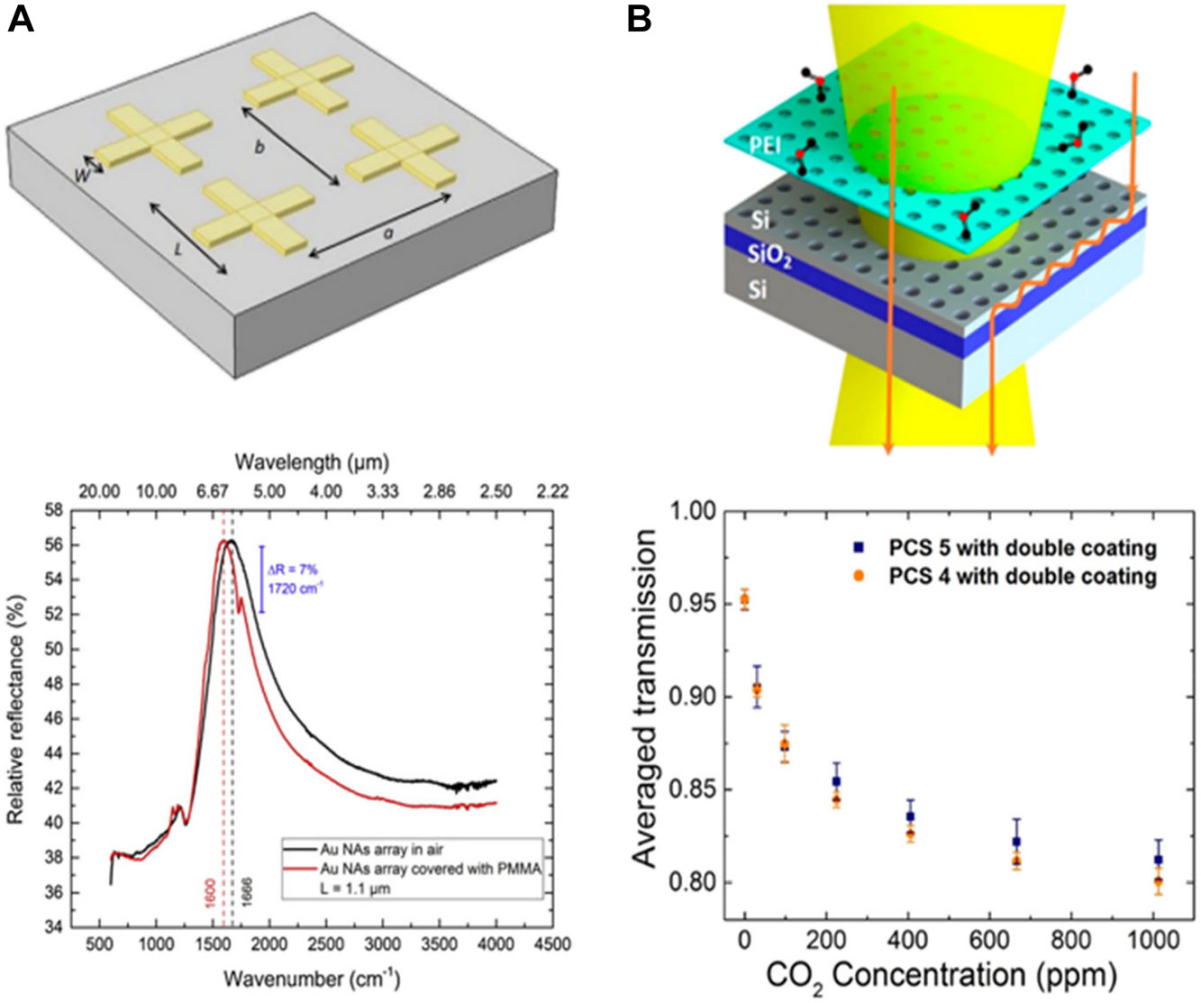
SEIRA based optical sensor. (A) Truncated view of the nanoscale antennas (NAs) 2D-array (top). Measured reflectance of the NAs-array is shown: the black and red curve are referred to bare NAs-arrays and NAs-array covered with a 50 nm PMMA layer respectively (bottom). Adapted with permission from ref. [24], copyright 2019, Elsevier. (B) Illustration of SEIRA based gas sensor (top) and measured transmission of two types of photonic crystal slab (PCS) (bottom). Adapted with permission from ref. [25], copyright 2018, American Chemical Society.

IR wavelength is the most widely used band including commercialized equipment to date from a spectroscopic point of view as it contains abundant information on the vibrational mode of atoms and molecules. The functional groups in molecules for instance vibrational bands can thus be directly characterized in non-destructive and label-free manner. With the advantages, chemical compounds identification, cancer identification, and discrimination of bio/chemical species were broadly investigated. However, low molecular absorption cross-section at IR (*σ*_abs_ ≈ 10–20 cm^2^) still prohibits the sensing of trace molecules in a low concentration. To assist the sensing efficiency, surface enhanced infrared absorption (SEIRA), which originated from the LSPR phenomenon, was suggested. Cross-shaped nanoantenna-array revealed mid-IR response enhancement up to 104–105. Also, freely tunable resonant plasmonic response can be matched to the carbonyl group vibrational band directly (1660 cm^−1^) (Figure 5A) [24]. By means of the enhanced absorption, tiny molecules in a very low density were observable. Meanwhile, in order to minimize ohmic loss and strong heating inevitably accompanied when using a metallic plasmonic structure, loss-less all-dielectric nanostructure was suggested as alternatives with comparable field enhancement and high *Q*-factor. The excited guided resonance by a periodically patterned silicon slab can selectively react with the fingerprint of CO_2_ gas with detection limit of 20 ppm (Figure 5B) [25].

Recently, various types of metasurfaces that boost high efficiency and variety of functionality in the terahertz (THz) regime have been developed, spurring the commercialization of THz technology [30, 31]. Considering its short history for biochemical sensing, the fast-growing THz technology promises huge potential for satisfying the needs of the medical science and health industry within the next few years. Unlike X-ray, which has a share of the biggest medical imaging market as well as medical science, the THz electromagnetic wave is non-ionized and non-destructive that does not cause damage to tissues or DNAs [32, 33]. In addition, since huge water absorption of the THz wave provides clear contrast information depending on the presence or absence of water, it is useful for bio-sensing and -imaging counterintuitively as it received great attention in early 2000s. Since there are intrinsic vibration modes of various molecules at broad bands of THz regime such as IR, their fingerprint frequency information can be directly used for sensors. Considering that key factors for judging performance of the sensor are ‘sensitivity’ and ‘selectivity’ of detected molecules, in this respect, THz wave has excellence to discriminate and identify the target molecules as compared to other wavelength regimens. IR spectroscopy and Raman spectroscopy employ fundamentally detection principles; molecular fingerprint frequencies determine molecular species. However, given that the THz band is a few orders wider, it is much more likely useful as avoidable vibration overlapping of inter- or intra-atoms or molecules.

**Figure 6:**
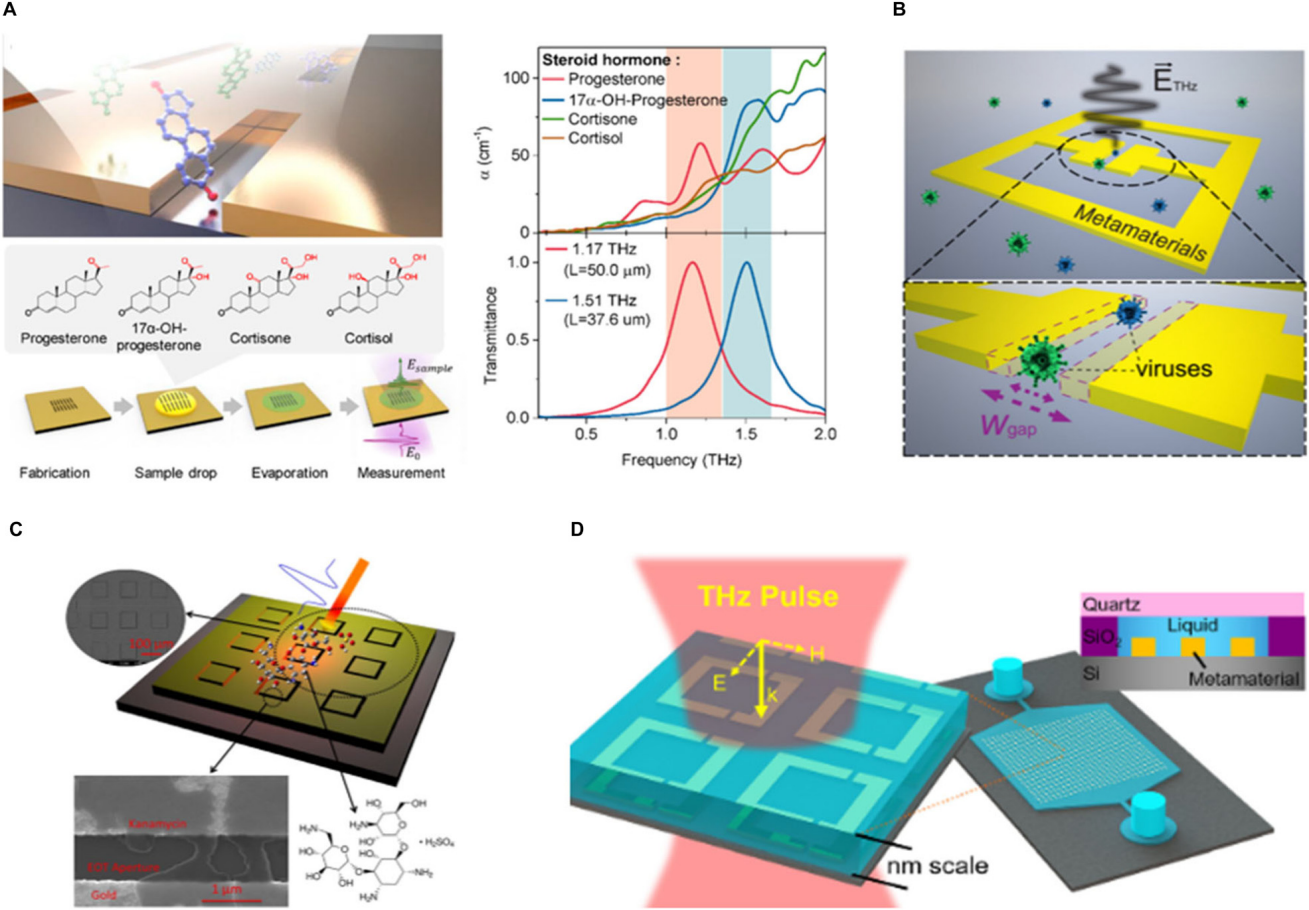
Highly selective THz metasensor. (A) Schematic of steroid sensing using THz nanoslot sensing (left). THz absorption spectra of steroid hormones in pellet types and transmission spectra of nanoslot-arrays (right). Reproduced with permission from ref. [26], copyright 2019, American Chemical Society. (B) Schematic diagram of THz nanogap metasurface sensing of dsDNA and single-strand RNA viruses. Reproduced with permission from ref. [27], copyright 2017, Optical Society of America. (C) Diagram of metasurface-assisted THz sensing of kanamycin sulfate. Reproduced with permission from ref. [28], copyright 2015, Springer Nature. (D) Illustration of the nanofluidic THz metasensor and its cross-sectional device structure. Reproduced with permission from ref. [29], copyright 2018, AIP Publishing.

Despite these various advantages, however, THz sensing and imaging bio-applications, have stagnated the development of THz sensors due to too low absorption cross-section of molecules at these regimes causing significant limitations of LOD. The remarkable growth of metasurface technology gratefully improved the sensitivity at a specific frequency, getting over the limitation. Efficient molecular-specific sensing can be established with the THz metasurface sensing chip, and the functionality can be further expanded by dynamical control of the resonant frequency and responses. Steroid hormones which have unique absorption features at THz regime can be discriminated by using a differently resonant nanoslot antenna-array (Figure 6A). For such performance, fingerprinting through the pellet type of samples were performed and then the resonance frequency was selected to match the target’s fingerprint. The dropped samples can be then observable even in a few molar concentrations. By extremely enhanced absorption cross-section using nanoslot-array designed for steroid fingerprint, only 9 ng of it was achieved with simple drop and dry technique [26]. By exploiting split-ring resonator metasurface, bacteriophage viruses PRD1 (60 nm, dsDNA) and MS2 (30 nm, ssRNA) detection were well accomplished and complex dielectric constants of virus were obtained (Figure 6B) [27].

Antibiotics, which is an antimicrobial substance acting against bacteria infection and pathogenic microorganisms, is widely used for treatment and prevention of infection. However, severe side effects associated with allergic reaction, organelle damage and bacterial resistance became issued in recent years. Tiny amount of kanamycin sulfate antibiotic material was sensitively detected with THz spectroscopy via metasurface with the square-shaped metal patterns. By using metasurface, the lowest detectable quantity was 100 pg/L which is 10^10^ times magnified than that without the metasurface (Figure 6C) [28]. Few studies attempted to confine aqueous solution surrounding metasurface below micro scale. Confining the solution into extremely small size using a microfluidic system not only be able to reduce the liquid absorption, but also deliver the bio-molecule efficiently to sensing hotspot. Lee et al. demonstrated the nanoscale (160 nm) confined microfluidic THz metasurface sensing platform which shows remarkable sensing of adenosine triphosphate (ATP) (Figure 6D). ATP aptamer functionalization on the surface of gold was additionally adopted, then Fano-type resonator demonstrated efficient detection of the ATP with the lowest detection concentration of 0.1 μM [29].

## 3 Precise manipulation of particles via field induced by micro/nano architecture

**Figure 7:**
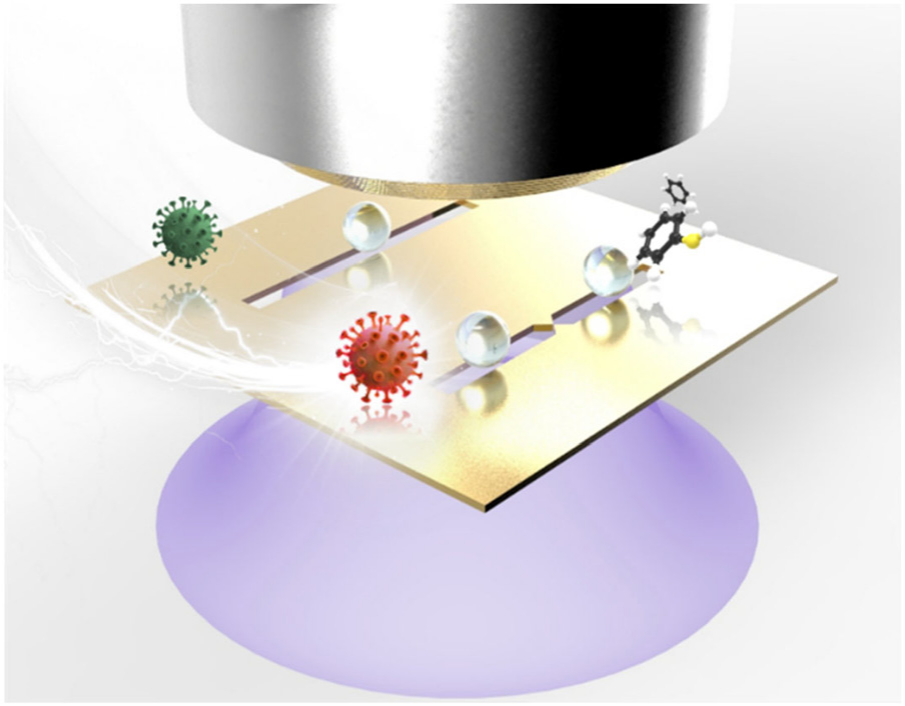
Schematic illustration of photo-electric tweezing platform of nanoparticles and biochemical substances using plasmonic metasurfaces. The critical role of the photo-electric tweezing platform is based on the large-scale plasmonic structures to trap the target particle/molecules and detect the targets simultaneously.

Precise manipulation of extremely small molecules and their assemblies (in forms of nanoparticles) has recently become a prerequisite technology especially in the field of nano-biosensors. Despite their increasing demand in wide ranges of studies and industries, control of nanoparticles is still challenging owing to the disruptive Brownian noise. A variety of particle manipulating techniques were developed using microfluidic-, acoustic-, mechanical-, magnetic-, optical-, plasmonic- and electrical stimuli. Among them, plasmonic and electrical approaches have become more suitable candidates for development of nano-biosensors thanks to its active and easy actuation in a remote manner as well as uniform applicability over large areas for massive particle controls (Figure 7). They are highly advantageous than the others for particle manipulation in nano scale precision in that these techniques rely on an induced field by micro/nano architecture. In this chapter, theoretical principles of plasmonic and electrical tweezing technologies are extensively discussed and monumental experimental studies are reviewed.

### 3.1 Plasmonic nano-tweezer

Ever since introduced by Ashkin in 1970 as ‘optical tweezers’ [34], particle capture via focused laser beam has become one of the most frequently conducted particle manipulation methods. Its uniqueness on direct capture and transportation of individual particles toward an optically activated zone of interest attracted huge intentions from fundamental physics, applied chemistry to medical- and biological science. Despite such superb initiation of new technology, however, optical trapping via far-field laser focusing under microscope objective lenses with high numerical aperture accompanies fundamental limitations. First, the optical diffraction limit hinders high precision manipulation since it requires minimum beam spot size to be on the order of wavelength. Second, usage of high power lasers is inevitable for optical trapping of extremely small nanoparticles (1.5 W for 9–14 nm [35]). Irradiation of such high power laser beams may lead to possible damage to target particles, especially for biological specimens. Third, strong light reflection, scattering, and absorption on metallic bodies make the optical tweezing of metal nanoparticles even more challenging. As an alternative, particle trapping principles via near-field plasmonic optical tweezers were introduced and developed to compensate for direct laser-irradiated trapping schemes via far-field optical tweezers.

The principles of plasmonic trapping follow general optomechanics of optical trapping. For particles of Mie scattering condition, that particle radius (*r*) is much larger than wavelength (*λ*) of incident light (*r* ≫ *λ*), an optical force arises from refraction on particles and can be calculated by geometrical ray optics [36]. On the other hand for Rayleigh regime of much smaller particles than wavelength (*r* ≪ *λ*), particle can be approximated into a point dipole by effective dipole moment theory. For theoretical understanding, time-averaged optical force (⟨**F**⟩) acting on a point-dipole particle under electromagnetic fields (**E** for electric field and **H** for magnetic field), can be described by two dominant components [37], ⟨**F**⟩ = (1/4)Re[*α*]∇**E** ^2^ + (*σ*/2*c*)Re[**E** × **H** ^*^], where *α*, *σ*, and *c* denote polarizability of particles, extinction cross-section, and speed of light respectively. Each component indicates *E*-field intensity gradient (first term) [35] and radiation pressure (second term) [34] which are associated with gradient force and scattering force respectively. The gradient force, which arises from inhomogeneous external fields, gives rise to translational motion of particles in direction of spatial field distribution gradient and confines particles inside the optical trap. On the contrary, scattering force arises from absorption/re-radiation on the particle and migrates particles in direction of light propagation. Therefore, optical trapping of a particle occurs when gradient force is dominant over scattering forces and generally requires potential depth to be 10 *k*_B_ *T* (*k*_B_ and *T* note for Boltzmann constant and absolute temperature) for stable tapping over the particle’s thermal energy [35].

**Figure 8:**
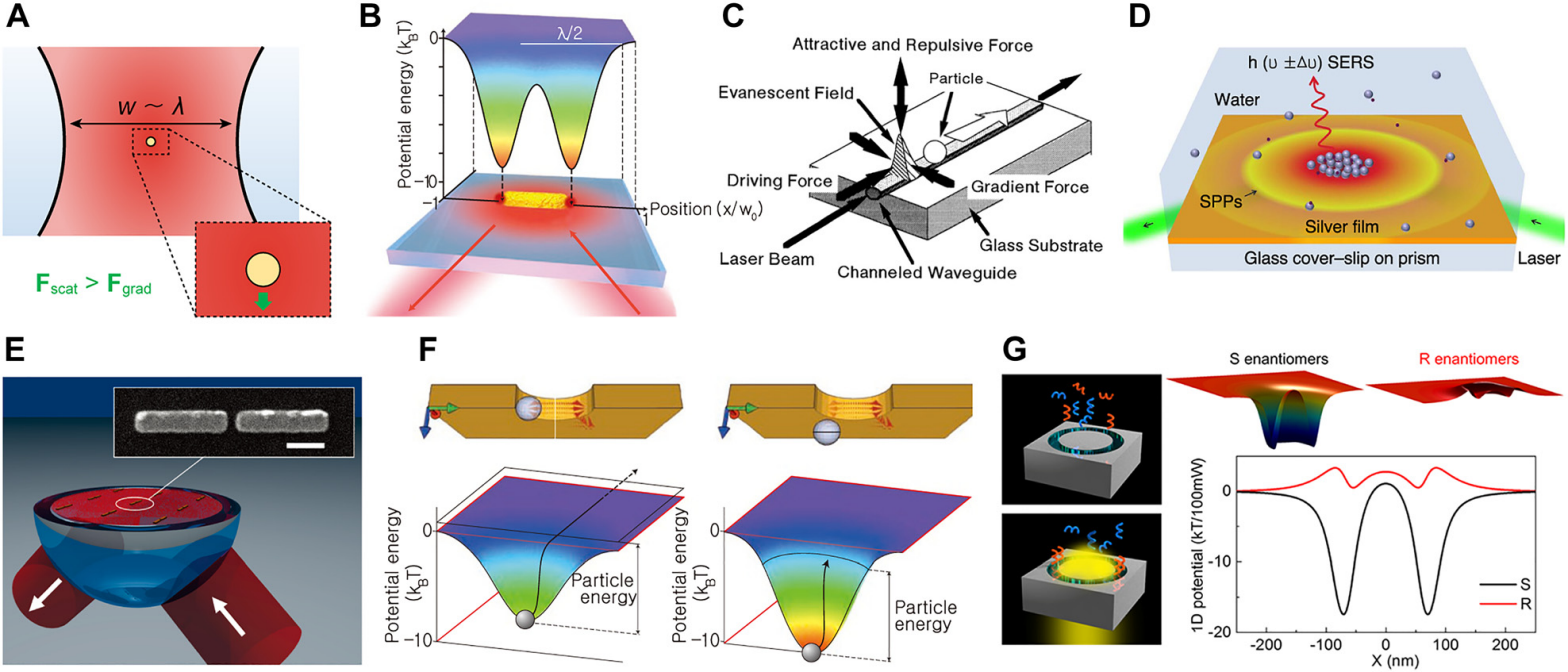
Principles and applications of plasmonic particle tweezing. (A) Diffraction limit on optical trapping by far-field focusing. (B) Plasmonic trapping on gold nanorods and its potential energy landscape. Reproduced with permission from ref. [38], copyright 2011, Springer Nature. (C) Migration of particle by excited SPP evanescent field. Reproduced with permission from ref. [39], copyright 1996, Optical Society of America. (D) SPP plasmofluidic ordering of nanoparticles. Adapted with permission from ref. [41], copyright 2014, Springer Nature. (E) Plasmonic trapping of *Eschetichia coli* bacteria on metallic nanorods. Reproduced with permission from ref. [42], copyright 2009, American Chemical Society. (F) Effect of SIBA restoring force on augmenting plasmonic trapping potential depth on metallic nanoaperture. Adapted with permission from ref. [38], copyright 2011, Springer Nature. (G) Enantio-selective chiral plasmonic trapping on annular aperture. Reproduced with permission from ref. [43], copyright 2016, American Chemical Society.

In the far-field focusing of the laser, diffraction limit defines minimum scale of beam spot to be in the order of wavelength. Henceforth, when a particle much smaller than wavelength is located in the focused far-field, the particle regards the optical field as a homogeneous wave (Figure 8A). Since spatial field gradient is significantly small in this case, scattering force becomes dominant over gradient force, limiting optical trapping. Here, optical trapping by intensified near-field on plasmonic nanostructures, referred as plasmonic trapping or plasmonic tweezer [38], can be an excellent alternative. Since confined near-field on an optical hotspot is highly associated with the geometry of the tweezers rather than wavelength, subwavelength plasmonic nanostructures can produce strong and stable trapping forces for Rayleigh particles (Figure 8B). In this case, therefore, scales of particles become comparable to the characteristic length of confined electromagnetic waves and subwavelength particle trapping can be conquered.

Following the aforementioned theoretical prediction on the idea of integrating nanoplasmonics with optical trapping, experimental studies at the first stage presented plasmonic forces by SPP propagation on a thin planar metallic layer. When momentum matching condition is satisfied on a smooth metallic layer coupled with prism in Kretschmann geometry, SPPs are excited at metal-dielectric interface over an illuminated area and evanescent field of SPP propagation creates optical forces. Kawata et al. first reported accelerated migration of Mie particles along the direction of scattering force generated from SPP evanescent field [39] (Figure 8C). However, for trapping and confinement of particles, thermophoretic convection by SPP excitation is generally utilized rather than gradient force. Since convective flow by thermophoresis pulls particles toward the regions where temperature is higher, thermophoretic convection compensates scattering force in opposite directions. Enhanced optical forces in combination of thermophoretic convection, Garcés–Chávez et al. first observed arrangement of 5 μm silica beads over large scale [40] and Patra et al. recently exhibited SPP plasmofluidic ordering of 40 nm silver-gold nanoparticles on a plane metal surface in similar manner (Figure 8D) [41].

However, SPP-assisted trapping has a fundamental limitation in high-precision manipulation since it operates over a large illumination area. Moreover, plasmonic thermal effect prevents biological applications since it can thermally damage the target biomaterials. Instead, on the basis of theoretical prediction on trapping of dielectric particles on plasmonic metallic tips [44, 45], subwavelength optical traps with sufficiently strong gradient forces were suggested with nanoscale accuracy under non-focused illuminations and reduced laser intensity. As an effort to optimize trapping capability, plasmonic architectures were scale-downed to subwavelength-regime and implemented with alternative design configurations, which can be classified into two large categories; isolated nanoislands and perforated nanoapertures (detailed in [46]). After studies of Quidant’s group on experimental demonstration of trapping micron polystyrene (PS) beads (4.88 μm) on micron-scaled (4.8 μm) gold disks [47, 48], numerous designs of subwavelength isolated nanoislands (single-hole, double-hole, bow-tie, annular ring, and cavities) were presented overcoming limitations of trapping based on SPPs. For instance, Righini et al. demonstrated bio-applicability of plasmonic tweezers by *E*. *coli* bacteria aligned with the long-axis of nanorods (Figure 8E) [42]. For reducing photodamage, 800 nm wavelength laser was utilized which has comparably low photon energy in visible regime. A step further, plasmonic potential trap facilitates complex optical manipulation of particles such as sorting and conveying of particles. While Roxworthy et al. demonstrated size-dependent sorting of 0.5 μm and 1.5 μm PS beads on bowtie nanoantennas [49], and Hansen et al. proposed optical conveying of particles on designed arrays of C-shaped nanostructures [50]. On the other hand, for perforated nanoapertures, automated positive feedback can be exploited for enhanced trapping efficacy. Juan et al. experimentally corroborated that optical trapping on metallic nanoholes provides positive feedback to trapping force so it is named self-induced back-action (SIBA) optical trapping [51]. Local change in the surrounding refractive index at the vicinity of nanoaperture affects the capturing capability (Figure 8F). As dipolar interaction results in spectral shift or transmission increase, monitoring of trapping events is also possible with a variety of nanoaperture designs (disk, rod, pillar, pyramid, and diabolo).

Angular momentum gives rise to rotational motion for non-chiral particles, and even selective manipulation for chiral particles depending on handedness is possible. With the fact that objects in anisotropic shape such as elongated nanorods and nanowires can be easily rotated and aligned in direction of field polarization [52]. Zhang et al. presented metallic nanowires trapped by SPP and rotated by changing polarization direction [53]. To realize the rotation of particles even in isotropic shape, chiral metasurfaces were utilized. For instance, Tsai et al. showed a rotation of isotropic particles by enhanced near-field vortex on a metallic layer with spiral slots [54]. Liu et al. inserted plasmonic spirals into disk-particles for the particle rotation [55]. Moreover, separation of targets including nanostructures/molecules with different chirality is viable. Zhao et al. designed enantio-selective nanoplasmonic trapping by homogeneous optical chirality along the ring channel of aperture under circular polarization (Figure 8G) [43]. On the one hand, electro-magnetic polarizability of the particle contributes to chiral components of optical forces (interact differently respective of handedness) whereas electric polarizability is relevant to achiral gradient force components (interact identically irrespective of handedness). Since total optical force acts on a chiral particle in either constructive or destructive sum of chiral/achiral forces, S- and R-enantiomers experience different optical trapping potentials under left-handed circular polarization. Several theoretical research on opposite chiral dynamics suggested possibilities for chiral-selective optical separation [56], still, practical realization is ongoing.

### 3.2 Electrical nano-tweezer

Electrical manipulation methods are highly advantageous owing to simple and facile experimental schemes, enabling manipulation of large numbers of particles in a real-time and reproducible fashion. Suspended particles under applied electric fields via direct current (DC) or alternating current (AC) voltages experience electrokinetic movements in liquid. Among them, DC electrokinetics such as electrophoresis (EP) and DC electro-osmosis (DCEO) have contributed particle manipulations for a wide range of nano/bio applications. Both techniques can move the particles of intrinsically charged or charged-material labeled objects since charged objects can be affected under the electrostatic mechanism. Besides, the smaller target particles become, the higher applied voltages are needed. On the contrary, AC electrokinetic processes including dielectrophoresis (DEP) and AC electro-osmosis (ACEO) do not require cumbersome labeling to capture/sort/move the suspended particles in liquids. Also, the bias-voltage can be much reduced by scaling-down the electrode gap. For such convenience and versatility, AC electrokinetic methods are rapidly becoming a major stream of particle controls in fields of bio/chemical sensing, microfluidics, and lab-on-a-chip systems.

**Figure 9:**
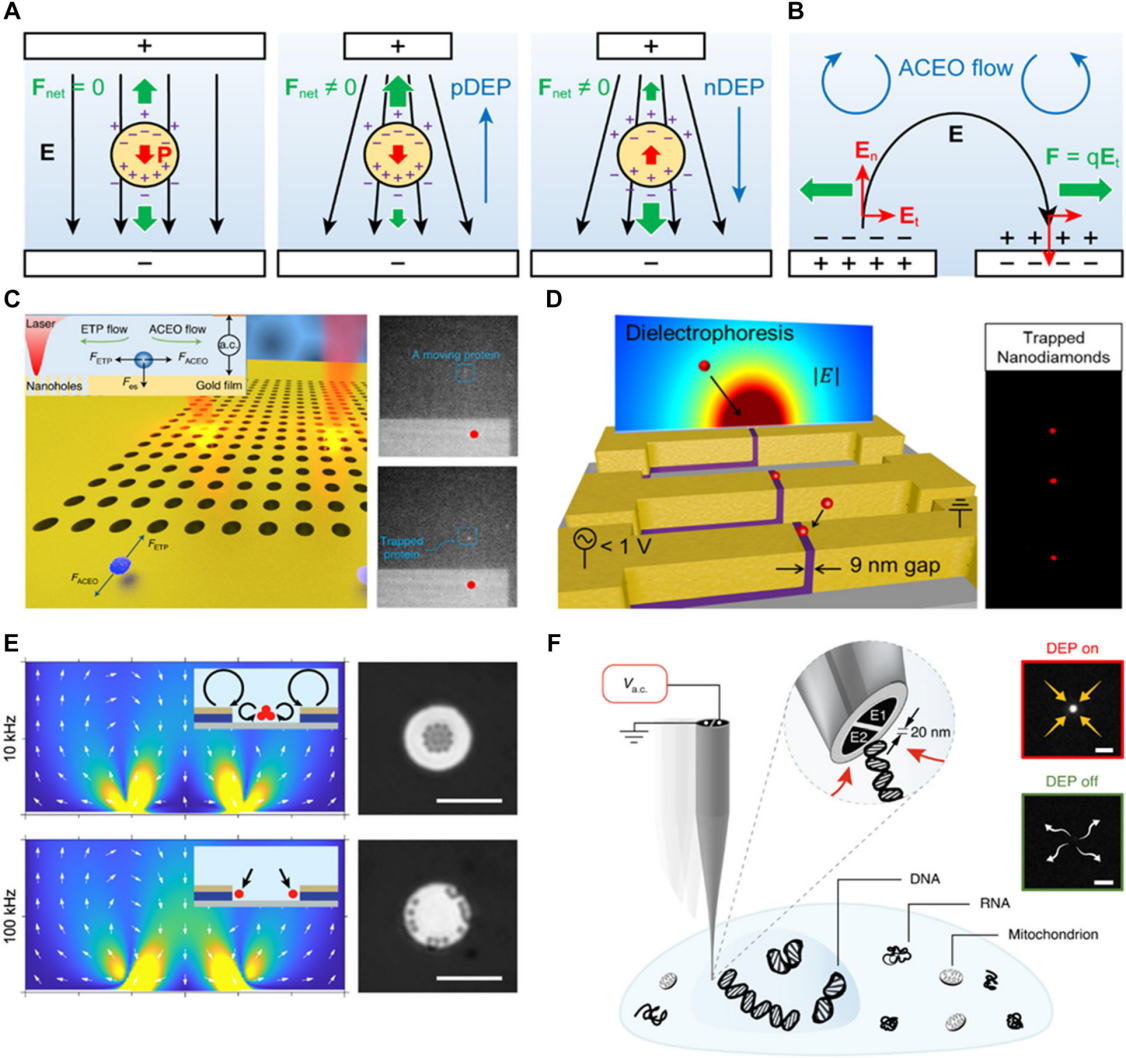
Principles and applications of AC electrokinetic particle tweezing. (A) DEP force acting on a particle under uniform- (left) and non-uniform *E*-field (right). (B) ACEO fluid motion on surface of potential applied electrodes [60]. (C) Particle trapping at stagnation zone of ACEO and thermophoresis on nanhole-array. Adapted with permission from ref. [63], copyright 2020, Springer Nature. (D) Dielectrophoretic particle trapping on nanogap and fluorescent microscopic image of trapped nanodiamond. Reproduced with permission from ref. [64], copyright 2016, American Chemical Society. (E) Simulations of particle dynamics on vertical nanogap electrode for combined DEP/ACEO for relocation of particles on the metasurface. Adapted with permission from ref. [61], copyright 2020, Springer Nature. (F) Dielectrophoretic nano-biopsy and fluorescent microscopic images of DNA trapping and releasing. Adapted with permission from ref. [65], copyright 2019, Springer Nature.

Discovered by A. Pohl et al. [57], DEP refers to the translational motion of polarized particles under AC volt-driven non-uniform *E*-field. Under applied *E*-field (**E**), charges within a dielectric medium move to the interface at which particle and medium contact. Regarding that field-induced particle polarization produces coulomb force, a neutral particle placed under uniform *E*-field experiences net force of zero. On the other hand, the polarized particle positioned under a non-uniform *E*-field experiences non-zero net force resulting in particle movements toward one of the electrodes (Figure 9A). By interpreting polarized particle as an induced dipole, the effective dipole moment theory derives time-averaged DEP force (⟨**F**⟩) acting on a spherical particle as [58], ⟨**F**⟩ = (1/2)Re[*α*]∇**E** ^2^. Particularly, *α* notes for polarizability of spherical particles; a parameter which characterizes polarizing behavior and dielectrophoretic polarity. For particles with higher polarizability than the surrounding medium (Re[*α*] > 0; positive DEP), the particle moves and becomes trapped where the local *E*-filed has maximum (electrical potential has minimum). In contrast, particles with lower polarizability (Re[*α*] < 0; negative DEP) are repelled from maximum to maximum position of the local *E*-field. Dielectrophoretic force dependent on the volume and electric property of particles can be further investigated in terms of various size and conductivity of the particles.

When a voltage is applied on an electrode immersed in electrolyte solution, oppositely charged ions are gathered and accumulated atop the electrode surface as they form an EDL. Based on Helmholtz–Smoluchowski theory [59], interaction between counterions within the EDL and tangential components of *E*-field generates electric forces, inducing lateral motion of ions and fluid flow from edge-to center of electrodes. Consequently, this leads to creations of continuous long-range flow over the electrode system (Figure 9B). The electrohydrodynamic flow arising from non-uniform AC *E*-field was discovered by Ramos et al. and denominated as an ACEO [60]. Since ACEO phenomena occur on the surface of electrodes, especially, right above the EDL, the control of particle movements over the electrodes can be accomplished by tuning the ACEO velocity. Tuning such values (i.e., solution conductivity, AC voltage amplitude, and frequency) can transfer the particle of interest using ACEO. As recently reported, DEP and ACEO is the dominant electrokinetic phenomenon, manipulating the suspended particle in the medium [61, 62]. Thus, the other external forces such as electro-thermal effect, gravity, buoyancy, and particle–particle interaction, will not be seriously counted in this review.

While ACEO delivers particles from a distant place, DEP migrates and traps them in a specific area. Studies in the fields of ACEO have mainly focused on establishing ACEO-assisted ‘microfluidic pumps’ for generation of directional net flow to transport or convey the particles. Directional ACEO flow was controlled by employing different physical conditions such as electrode widths [66], orientations [67], and three-dimensional configurations [68]. Applying asymmetric electric signals (DC bias [69], and traveling-wave *E*-fields [70]) also contribute to originate directional flow by the ACEO phenomena. Hong et al. recently introduced integration of ACEO with thermo plasmonic force for trapping of sub-10 nm molecules from femtomolar concentration (Figure 9C) [63]. From thermal and electric field gradient components, two opposing convective flows of thermophoresis and ACEO are generated inward and outward center of the array, giving rise to a stagnation zone where the flow velocity becomes zero. In this manner, a single BSA molecule from 15 fM can be trapped at the stagnation point.

For trapping of extremely small nanoparticles down to sub-100 nm, conventional DEP electrodes with a micron scale gap distance require high amplitude of voltages and this causes undesirable flow, bubble, and Joule heating. To avoid those issues, an extremely small gap distance or sharp edge in electrodes was suggested generating sufficiently strong *E*-field even under low voltage bias. Along with the rapid development of nano-fabrication techniques, electrode with gap distance of 5–150 nm was fabricated for trapping of 30–120 nm particles and DNA molecules at AC peak-to-peak voltages (*V*_pp_) up to a few volts in the ranges of 2–4.5 V [71], [72], [73], [74], [75], [76]. For the sub-volt DEP tweezing, Oh’s group introduced sub-10 nm in-plane nanogap fabricated by atomic layer lithography [64] and angstrom-scaled electrode edge by exploiting single graphene as electrode on vertical stack architecture [77] (Figure 9D). In these works, nanoparticles with their diameters of 40–190 nm and DNA molecules were captured with minimum AC peak amplitude (*V*_pk_) of 0.3–0.6 V and 2.5 V, respectively. Yu et al. demonstrated comparable high precision electric tweezing performance (trapping 50 nm particles by AC *V*_pp_ of 0.33 V) with frequency-dependent particle relocation by sub-100 nm vertically-aligned electrodes (Figure 9E) [61]. In this work, it has been numerically corroborated that higher *E*-field gradient (∇*E* ^2^) was generated at the vertically-aligned electrodes than the horizontally aligned electrodes under identical gap distance. Very recently, the most improved DEP manipulation of biological molecules to a level of single-molecule was exhibited by Edel’s group. Simply fabricated nanopipettes with 10–20 nm gap extracted a single-molecule of DNA, mRNA, and organelle from cells [65] (Figure 9F). Moreover, by coupling dielectrophoretic trapping with simultaneous electro-optical sensing modalities, the highest single-molecular sensitivity was accomplished (detecting a single-molecule from concentration of 5 fM [78] and 0.1 fM [79]) with capabilities to detect protein binding [80] and differentiate individual protein sizes [81].

## 4 Metasurface sensors combined with directed transport for overcoming diffusion limit

For a decade, an enhancement of the electromagnetic field on the metasurfaces has contributed to development of simple, rapid, and robust on-site molecular analytic devices. Majority of metasurface-based sensing methodologies generally rely on amplified optical signals initiated by light–matter interaction. Electromagnetic waves can be extremely confined and maximized at the nanoscale volume of the specific region which is widely defined as optical hotspots. Thus, concentration of the target molecule at the optical hotspots is one of the key criteria for developing efficient analytical sensors. A variety of demonstrations has been introduced promoting transportation of targets into optical hotspot sites, which is generally defined as ‘guided transport’ [82]. Else, under the absence of such guided transportation, molecular random walk by free diffusion becomes the only driving mechanism for localization of target analytes into the detection site. This issue becomes more problematic for the detection of extremely low concentrated molecules. For example, it is theoretically examined that the process of diffusive accumulation of 1 fM target analytes toward the surface of sensors takes several hours to a few days [83]. This chapter discusses various approaches to overcome diffusion limits of nano scale metasurface-based biosensors through guided transport strategies. In the review, we broadly classified them into ‘self-guided passive transport’ and ‘field-induced active transport’.

### 4.1 Self-guided passive transport

**Figure 10:**
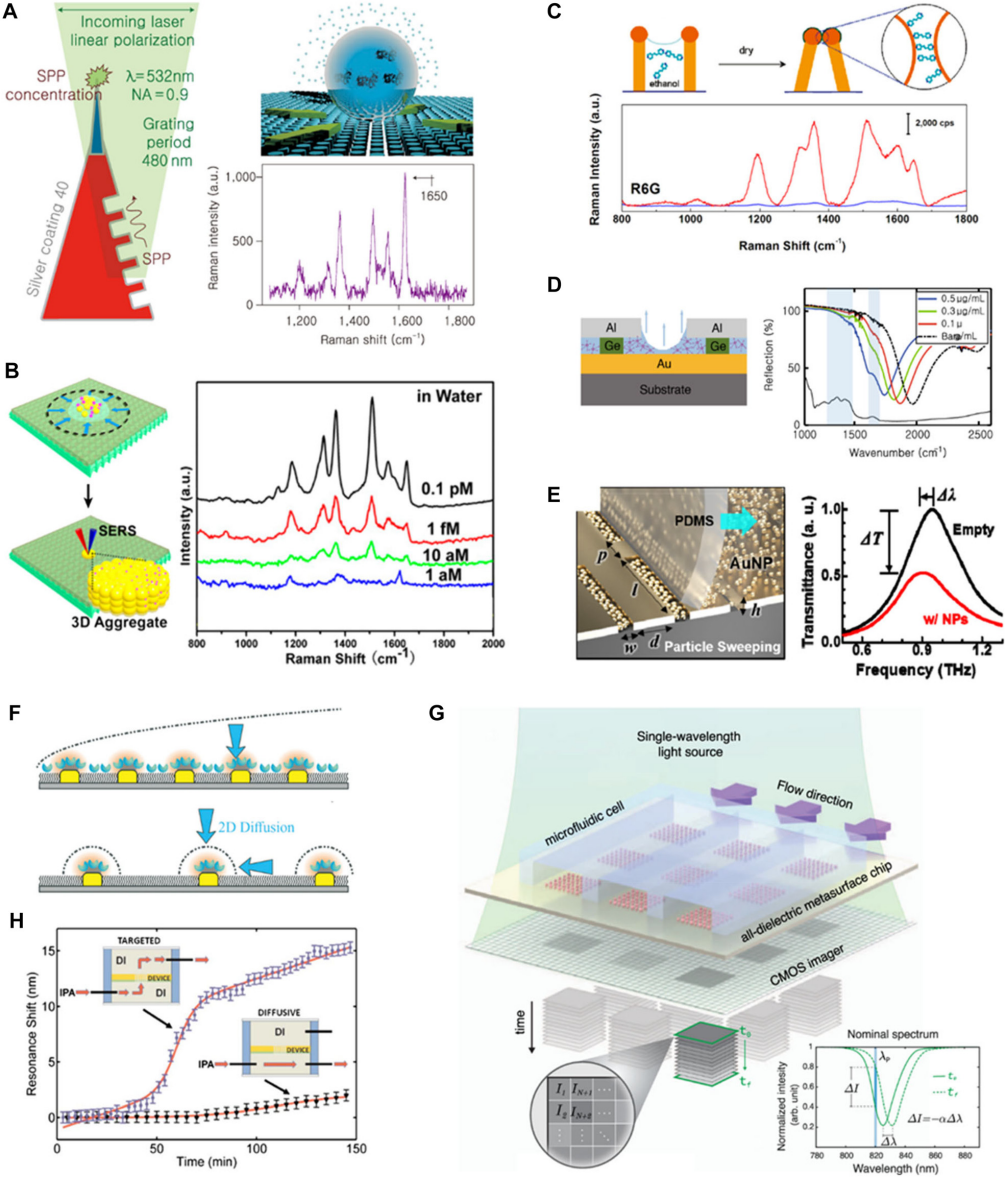
Surface-enhanced sensing on metasurface combined with self-guided passive transport. (A) Evaporation-induced molecule concentration over the plasmonic focusing nanocone and Raman spectra for detection of 1 fM lysosome. Adapted with permission from ref. [84], copyright 2011, Springer Nature. (B) Evaporation-induced molecule concentration and Raman spectra for detection of rhodamine 6G on hydrophobic SERS platform. Adapted with permission from ref. [85], copyright 2016, National Academy of Sciences. (C) Capillary force-driven nanostructure clustering for achievement of enhanced optical signals. Raman spectra for detection of rhodamine 6G on mushrooms before and after clustering. Adapted with permission from ref. [87], copyright 2010, American Chemical Society. (D) Evaporation-assisted molecule concentration at the trench of ribbon-resonator. Adapted with permission from ref. [90], copyright 2021, Springer Nature. (E) Mechanical sweeping for concentration of gold nanoparticles into THz nanoslot. Adapted with permission from ref. [91], copyright 2017, Optical Society of America. (F) Molecular depletion zone and diffusion on plasmonic sensor with uniform- (upper) and orthogonal functionalization (lower). Adapted with permission from ref. [92], copyright 2019, Royal Society of Chemistry. (G) In-flow imaging platform with diatomic all-dielectric metasurface for extracellular vesicle detection. Reproduced with permission from ref. [97], copyright 2021, Springer Nature. (H) Time-lapse spectral shift for detection of isopropyl alcohol on nanoholes by flow-over and flow-through strategies. Reproduced with permission from ref. [101], copyright 2010, AIP Publishing.

As an effort to concentrate target analytes atop the signal hotspots, evaporation-driven molecular transportation was proposed with help of surface pretreatment techniques in a hydrophobic or a hydrophilic manner. In the case of a non-wettable hydrophobic surface of nanogroove, a water droplet exists in Cassie state in order to decrease contact surface area against the hydrophobic surface; water droplet sits with air pockets, which are trapped underneath. Given this, strategic design of the optical hotspots in the vicinity of evaporation sites results in improved capability of molecule concentrating. Angelis et al. demonstrated evaporation-induced delivery of target molecules over the plasmonic nanofocusing surface enhanced Raman spectroscopic (SERS) structures by surrounding silver nanocone with hydrophobic micropillars [84] (Figure 10A). Excited SPP from the grating is focused toward the tip of the nanocone, allowing molecular accumulation at the tip. In this work, amplified Raman signals of molecules in extremely low concentrations (10 aM of Rhodamine 6G and 1 fM of lysozyme) were rapidly monitored within few minutes. Similarly, Yang et al. accomplished detection of Rhodamine 6G molecules in sub femtomolar level (75 aM) by liquid pinning and evaporation-induced delivery via hydrophobic nanotextured surface (Figure 10B) [85]. On nanorod antenna-array surrounded by hydrophobic pillars, SEIRA detection of Apoferritin proteins in 10 fM and identification of secondary *α*-helical structure were carried out through evaporation-guided accumulation. The antenna is designed to selectively enhance *E*-field at the vibrational modes of amide I and II from ferritin [86]. In vice versa, evaporation-induced sensing on optical nanostructures can often be accomplished by ensuring Wenzel’s wetting state; water completely wets the complex geometry of the surface. For example, wetting and evaporating the gold-capped mushroom-like structures result in highly sensitive SERS detection by concentration of capillary force-driven clustering and delivery of target analytes at the optical hotspots (Figure 10C) [87], [88], [89]. Miao et al. presented self-guided molecular accumulation into hotspot sites via evaporating droplet on a hydrophilic SEIRA nanoarchitecture composed of periodic ribbon metal-insulator-metal resonator [90]. As molecules (L-proline and D-glucose) are self-directed and trapped into undercut hotspot regions during evaporation proceeds, spectral changes of reflectance can be detected with an enhanced SEIRA sensitivity of 1 pg limit (Figure 10D). Besides, the evaporation-driven arrangement of particles with rigid bodies can be further promoted by utilizing mechanical forces during the evaporation process. Ryu et al. demonstrated THz detection of sub-100 nm gold nanoparticles by sweeping PDMS film over the surface of the nanoslot-array after drop-casting gold nanoparticle solution [91]. Fully occupied gold nanoparticles by the mechanical sweeping result in transmission reduction and resonant spectral shift of nanoslot-array, overcoming small absorption cross-section of terahertz regime (Figure 10E).

Microfluidics has also been integrated with photonic sensors owing to its huge potential for realization of a practical total-analysis systems. As well as for (i) breaking diffusion limit and enhancing detection performance, integration of the microfluidic system with photonic analyzing techniques is advantageous on (ii) ease of chip fabrication, (iii) real-time sample exchange, (iv) small amounts of sample requirement (microliter-to-nanoliter), and v) prevention of contamination. In order to enhance the sensing capability, site-selective immobilization of the receptors to the optically functional sites should be realized while others are passivated, called ‘orthogonally functionalized metasurface’ [92]. This is because of the formation of depletion zone where the local concentration of target analyte is lower than bulk region. While uniform surface of bioreceptors is subject to severe diffusion limitation, orthogonally functionalized optical-functional-sites quickly reaches into full target collection then improves detection capability by facilitating diffusion for recovery of localized depletion (Figure 10F). The recent studies of binding kinetics reported that rate of analyte transport on metasurface substrate (defined as mass transfer coefficient) is governed by optical and channel geometries as well as for volumetric flow rate [93]. Sreekanth et al. assembled a microfluidic system with grating-coupled HMMs which exhibits extremely high refractive index sensitivity of 10,000–30,000 nm/RIU and 590 FOM in near IR regime [8]. Using microfluidics and standard streptavidin-biotin affinity model, ultra-small biotin molecules (less than 244 Da) of picomolar concentrations were successfully probed by 4 nm spectral shift within 40 min. Especially, Altug’s group is highlighted for their various cutting-edge optical biosensing technologies combined with microfluidics including SEIRA spectroscopy [94] and imaging on pixelated all-dielectric metasurface [95], [96], [97]. For example, SEIRA configuration in the mid-IR regime, which enhances absorption signals by coupling vibrational modes of the analytes and optical resonance of plasmonic nanoantennas, has huge potential for biochemical sensing applications through comprehensive information of analytes. In this field of SEIRA, they suggested internal reflection techniques combined with microfluidic technologies for overcoming severe absorption of liquid water in the mid-IR regime, which has remained long-standing challenge [94]. Besides, pixelated diatomic all-dielectric metasurfaces were proposed for improved detection and imaging of absorption fingerprints of target molecules in mid-infrared [95] and near-infrared regime [96]. Utilizing changes of transmission intensity from the binding of biomolecules, spectrometer-less imaging and detection of breast cancer extracellular vesicles were accomplished in real-time in 204 fM concentration (Figure 10G [97]).

Going beyond the flow-over the photonic nanoarchitectures, flow-through nanohole-array was employed as nanochannels [98, 99]. As mentioned in chapter 2, plasmonic nanohole structure enables EOT in several orders of magnitude larger and thus, applied to wide ranges of label-free biosensing [100]. Furthermore, compared to the diffusive delivery on the conventional flow-over channel that flows tangentially over the surface of optical nanostructure, perpendicularly steered flow toward the surface on flow-through nanohole significantly improves transport rate and sensitivity. Figure 10H showed that flow-through nanoholes improved the detection limit of plasmonic biosensing by more than 2-fold accompanied by increased mass transport rate and reduced detection time [1010].

### 4.2 Field-induced active transportation

Despite remarkable achievements in development of analytical devices, passive methodologies inherently possess limitations in dynamic molecular detection under liquids. While microfluidic-biased molecular detections utilize dynamic and real-time molecular monitoring underwater, it suffers from limited sensing capability by the formation of the molecular depletion zone [102]. Evaporation-induced transport leads to the creation of drastic improvement of molecular detection from highly diluted molecules in liquids, however, it undergoes exposure of target molecules in air which occasionally causes denaturation of biomaterials of the interest. In this situation, the plasmonic/electrical field-induced molecular detection can be an excellent alternative owing to its potential for active concentration of molecules toward the optical hotspots under aqueous environment as it opens a new avenue for establishing advanced surface-enhanced sensor devices.

**Figure 11:**
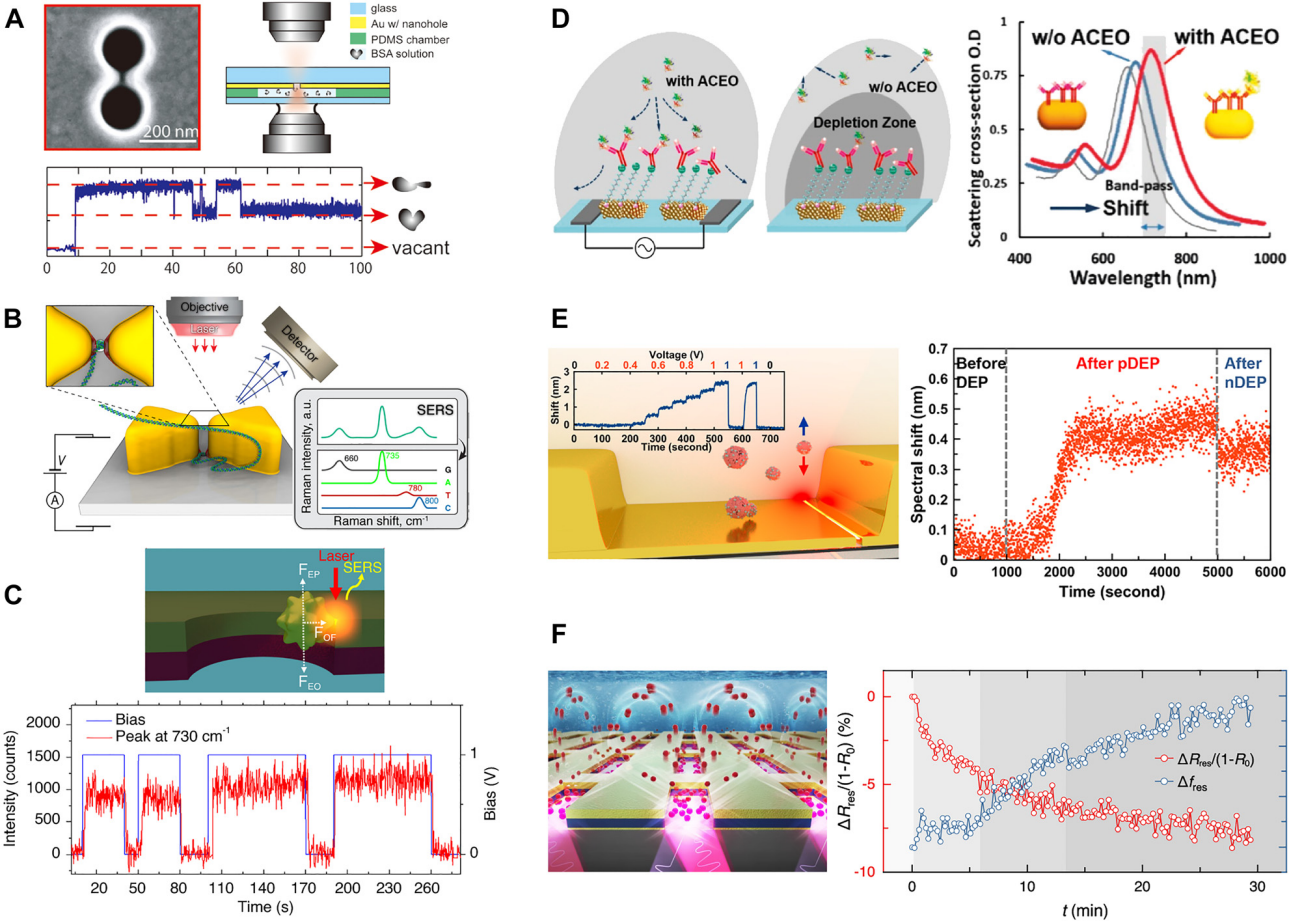
Surface-enhanced sensing on metasurface combined with field-induced active transport. (A) BSA protein trapping on double-nanohole and time-lapse monitoring of optical transmission intensity for detection of protein folding. Adapted with permission from ref. [104], copyright 2012, American Chemical Society. (B) Plasmonic nanopore bowtie for trapping and Raman sequencing of DNA molecule. Adapted with permission from ref. [112], copyright 2015, American Chemical Society. (C) Gold nanoparticle trapping at nanohole and real-time monitoring of Raman spectra for detection of DNA bases. Adapted with permission from ref. [119], copyright 2019, Springer Nature. (D) ACEO-enhanced gold nanoparticle gathering for improved scattering intensity for protein detection. Adapted with permission from ref. [123], copyright 2017, American Chemical Society. (E) Nanoparticle trapping on split-trench resonator and time-lapse monitoring of transmission spectral shift for detection of nanoparticle. Adapted with permission from ref. [126], copyright 2021, American Chemical Society. (F) Particle concentration into nanoslot by combined DEP/ACEO and real-time monitoring of terahertz reflectance for detection of nano-vesicles. Adapted with permission from ref. [62], copyright 2021, John Wiley and Sons.

The metasurface-related biosensing platform can be intuitively integrated with the plasmonic trapping platform because both platforms and underlying skills rely on identical physical phenomena. For perforated optical nanoarchitectures, monitoring transmission signals through the apertures also enable the characterization of trapping events simultaneously, Stepwise increase of transmission signal against single-particle attachment can be monitored due to optical performance. This ultra-sensitivity together with selective detection contributed to quantifying the biochemical event at the trapping sites. For example, Juan et al. demonstrated dual trapping-sensing of 50 nm PS particles on 310 nm nanoapertures with significantly reduced incident laser power of 1.9 mW (1064 nm in wavelength) by the principle and efficacy of SIBA optical trapping [51]. Biological target objects were further implemented for their capabilities in developing *in-situ* optical biosensors. By double-nanohole gold apertures with two sharp tips separated by 15 nm, Gordon’s group demonstrated plasmonic trapping and single molecular biosensing with light in the visible to the near-IR regime even under low laser power (∼3 mW) (Figure 11A). On the basis of sensitivity increment compared with isolated single nanoholes, they accomplished identification of compositions of biomolecules [103] and explained the mechanism of folding [104] and binding [105] at the level of single-molecule. On coaxial annular apertures of 180 nm-diameter which exhibit first-order Fabry–Perot mode at near-IR regime, Yoo et al. traced transmission signal and successfully monitored single molecular trapping of streptavidin protein from the concentration of 190 nM under 4.7 mW laser within 3 min [106]. In addition to transmissive nanoapertures, various plasmonic architectures were employed for capturing and monitoring. By utilizing an optical setup incorporating two respective beams for trapping and probing, Zhang et al. captured gold nanoparticles in-between plasmonic dipole nanoantennas and recognized capturing events in real-time through resonance shifts [107]. Also, on a microcavity which is consists of two mirrors (one flat and the other concave) separated by 1 μm, Trichet et al. tracked trapping of 70 nm poly(methyl methacrylate) nanoparticles inside microcavity by shifts of the resonance frequency of modes [108].

Despite capability for probing of trapping events at hotspot sites, however, monitoring transmission signal change is not enough for realization of practical analytic biosensors which necessitate comprehensive information on compositions or structures of biomolecules. For this reason, surface plasmons were utilized to intensify nano scale fluorescence for biomolecule identification since it reflects photon emission from specific biochemical components [109, 110]. For example, DNA molecules labeled with fluorescence probes were trapped on plasmonic nanoaperture and monitored with 100 folds fluorescence enhancement [111]. However, the fluorescence-based approach is far from substantive biosensors since it requires a cumbersome pre-labeling process. Thus, plasmonic tweezers were incorporated with functional spectroscopies which provide molecular-specific information. Dekker’s group suggested a nanopore located in the gap between nanobowties as a platform for DNA sequencing that facilitates plasmonic trapping of single DNA (Figure 11B) [112], [113], [114], [115]. Based on the fact that this platform enables stepwise displacement of DNA through nanopore, sequential information of a single DNA was identified through SERS signals [112] as well as for other label-free optical sensing methodologies [114, 115].

In Particular, assembling plasmonic metal nanoparticles into aggregates by optical tweezers has been utilized as a powerful method for enhanced spectroscopy. When multiple metallic nanoparticles are located inside the optical trap, spontaneous aggregation of nanoparticles arises and induced particulate electromagnetic fields overlap with external fields to prompt spectral shifts and field enhancement for strong optical gradient forces. Svedberg et al. reported the creation of SERS hotspot sites by dimerization of 40 nm silver nanoparticles and 22-fold increased SERS intensity for thiophenol detection [116]. Additionally, Xu et al. substantiated that molecules nearby can be trapped inside the nanogap region of metallic nanoaggregates even under low power laser exposure owing to intensified plasmonic trapping forces from plasmonic particle interaction [117]. Under incident laser of 10 mW/μm^2^ on dimer and trimer of silver nanoparticles (polarization parallel to direction of particle alignment), depths of optical potential well for trapping of Rhodamine 6G molecules were calculated to be 4*k*_B_ *T* and 6*k*_B_ *T*, which shows potential for capturing nearby molecules. This strategy of controlling and assembling plasmonic nanoparticles was combined with metasurface architectures for dramatic improvement of sensing capability. For example, Hong et al. collected 50 nm gold nanoparticles on 100 nm-gap nanobowtie antennas by 10 mW laser, reducing gap distance into 2 nm for 40 fold increment on SERS detection signal from 100 pM Rhodamine 6G [118]. In the work of Huang et al., electro-plasmonic trapping was exhibited for the creation of SERS hotspot site by coupling gold nanoparticles to the sidewall of nanoholes and identified oligonucleotides of single DNA bases attached at the surface of nanoparticles (Figure 11C) [119]. The electro-plasmonic trapping, which hybridizes plasmonic trapping (12 mW laser) with electrophoresis and electroosmosis (1 V), provides controllable and efficient trapping behavior by precise manipulation of balance between three different forces. Besides, for direct optical trapping, the mechanisms based on local plasmonic heating have been suggested as a tool for aggregation of metallic nanoparticles for SERS applications. Jin et al. suggested photothermal convection flow by lithographic assembly of gold nanoparticles [120]. By utilizing random nanoislands substrates for local heating by plasmonic absorption, Lin et al. utilized optically induced thermophoresis for dynamic nanoparticle manipulation which generates local *E*-fields by light-induced thermal gradient field [121]. On a similar nanoisland substrate, Kang et al. demonstrated formation of microbubbles by local plasmonic heating and spontaneous assembly of gold nanoparticles at the bubble-substrate boundary [122].

For electrically induced active transport, drastic improvement for molecular detectability was recently reported using AC electrokinetics nanoparticle tweezing coupled with various optical detection techniques. To capture and localize nanoparticles of faraway toward the detection zone, high voltage application is indispensable for DEP application. In this case, continuous electrohydrodynamic transport by ACEO can be an efficient alternative. This is because the ACEO allows delivering particles under a low voltage as it rules out biomolecule damage or denatures by Joule heating. Song et al. utilize ACEO to promote enhanced binding events of antibody-analytes. Antibody-conjugated plasmonic gold nanorods were placed in-between microelectrodes and analytes were migrated into depletion zone by rotational motion of ACEO fluid as it increases scattering cross-section accompanied by change of scattering intensity profile with red-shift of plasmon resonance (Figure 11D) [123]. By employing IL-1*β* signaling proteins with low molecular weight (17 kDa) for plasmonic quantification, the study reported that LOD was increased for 100 folds with ACEO actuation (15.2 pg/ml to 158.5 fg/ml) under 0.001× PBS condition. Cheng et al. exhibited on-chip SERS identification of bacterias (*S*. *aureus*, *E*. *coli*, and *P*. *aeruginosa*) from human blood by electrokinetic concentration on the ring-shaped SERS surface [124]. Taking advantage of long-range ACEO collection of bacteria from the mixed red blood cells, selective dielectrophoretic tweezing of bacterias onto the circular electrode where SERS signals are destined to be generated was accomplished. The DEP particle tweezing was further applied to nanogeometric metasurfaces where an EOT will be made. For example, Oh’s group captured BSA to the edge of the gold nanoholes and monitored from ultralow concentration (1 pM) as it dramatically reduces detection time (8 h–8 min for 10 pM) [125]. Besides, the sub-10 nm split-trench resonator platform is utilized for simultaneous DEP trapping and for detection of nanometer scaled PS particles via nanoplasmonic approaches (Figure 11E) [126]. The SPP modes excited from the nanogap propagate back-and-forth from the reflection by the sidewall of the trench. By designing the local position of nanogap, an intensity of resultant signals that arose from either constructive or destructive interferences are maximized. Thus, transmission Fano resonance of resonator can be easily tuned by modulation of sidewall width as well as the location of gap inside trench resonator. Consequently, stepwise increment of spectral shift from the trapping of 30 nm PS particles enables the analysis of real-time dynamics of particle behavior at a single-particle level.

On the mid-IR Fano resonant asymmetric metasurface whose resonance originate from interference between two different electromagnetic eigenmodes (subradiant and superradiant resonances) [127], Kelp et al. captured live cells (HCT116 and A431) and simultaneously monitored absorbance at spectral position of Fano resonance and amide I, II vibrational modes for characterization of cells [128]. Very recently, Yu et al. integrated terahertz nanoslot metasurface with a dielectrophoretic tweezer for real-time underwater detection of 50 nm-diameter small unilamellar vesicles (SUV) from extremely low concentration (1 ppm) (Figure 11F) [62] on the basis of their previous work of sandwiched electrodes for nanoparticle tweezing technique [61]. The 500 nm-width vertically-aligned nanoslot antenna plays bifunctional roles to (i) maximize terahertz signal and (ii) to concentrate floating SUV into the optical hotspots. With a help of long-range ACEO collection, remote particles move to the vicinity of nanoslot and are captured into the optical hotpots leading to time-lapse monitoring of underwater terahertz molecular behavior from the ultra-low molecular populations.

Going further from biosensing on pre-defined fixed architectures, reconfigurable plasmonic architectures by simultaneous dynamic relocation of plasmonic nanomaterials were also reported. By concentrating metallic nanoparticles over the pre-defined domain, additional optical hotspots were newly added at the specific local areas, resulting in significant improvement of molecular detectability. Cheng et al. developed an advanced SERS system using the reconfigurable detecting mechanism. An ACEO flow together with negative DEP assisted nanoparticle concentration at the pre-patterned gold island, enabling to amplify Raman signal (5 fold higher than signal without silver nanoparticles) for identification of low-density of bacteria [129]. Also, Wang et al. demonstrated rapid and sensitive Raman detection of breast cancer protein (HER2) via antibody-conjugated SERS silver nanoshells on SERS immunoassay by 4-fold increased Raman intensity and reduced detection time (24 h–40 min for 1 ng/ml) than conventional SERS immunoassay [130]. For extremely small nanoparticles, however, dielectrophoretic tweezing on electrodes with microscale gap distance requires a high amplitude of voltage that accompanies severe side effects. As an alternative, Ertsgaard et al. acquired amplified Raman signals from 70 nm liposomes by trapping both liposomes and 70 nm SERS gold nanoparticles on the 10 nm-nanogap with a minimum peak amplitude voltage of 400 mV [131].

## 5 Conclusions and outlook

In this review, we introduced up-to-date progress in metasurface-based biosensor chips from designs, material characteristics, to corresponding performances for great development of analytical devices. The working principles, followed by chip fabrication methods and their photonic/electric functions were thoroughly reviewed at the diverse geometric property of the metasurfaces. In particular, metasurface-based biomolecular sensors using diverse approaches that aim to deliver and localize the target nano-objects from colloidal nanowires, nanotubes, nanobeads to biomolecules and their assembly – into optically activated region were thoroughly discussed for addressing the new trend of metasurface-assisted photonic sensors. In addition, latest fashions of photonic approaches for detection of nanomaterials using optoelectronics and optofluidics including plasmonics, DEP and ACEO were addressed.

In summary, a reporting new concept and design of metasurface photonic devices followed by extraordinary optical performance leads the trend of metasurface studies at an early stage. They focused on introducing state-of-the-art metasurface architecture or materials-related geometry, demonstrating the underlying mechanisms of enhanced working performance as photonic devices. Next trend was led by collaborative works with biomolecular or environmental material detection using conventional particle manipulation techniques. For example, a number of self-guided passive transportation methods or field-induced active transportation of molecules described above led to the next trend of the analytic sensor platform. Despite such substantial efforts for developing photonic biosensor platforms, still, several hurdles have remained to solve. Major challenges can be; (i) the intrinsic nature of water absorption significantly limiting the broadband molecular detection such as molecular fingerprinting, and (ii) the trade-off between sensitivity and measurable volumes by drastic signal decrease from the sensing hotspots. Thus, a new paradigm of photonic sensor that is able not only to rule out possible water absorption during the broadband molecular detection, but also to be free from a limited area of sensing zone is highly desirable.

**Figure 12:**
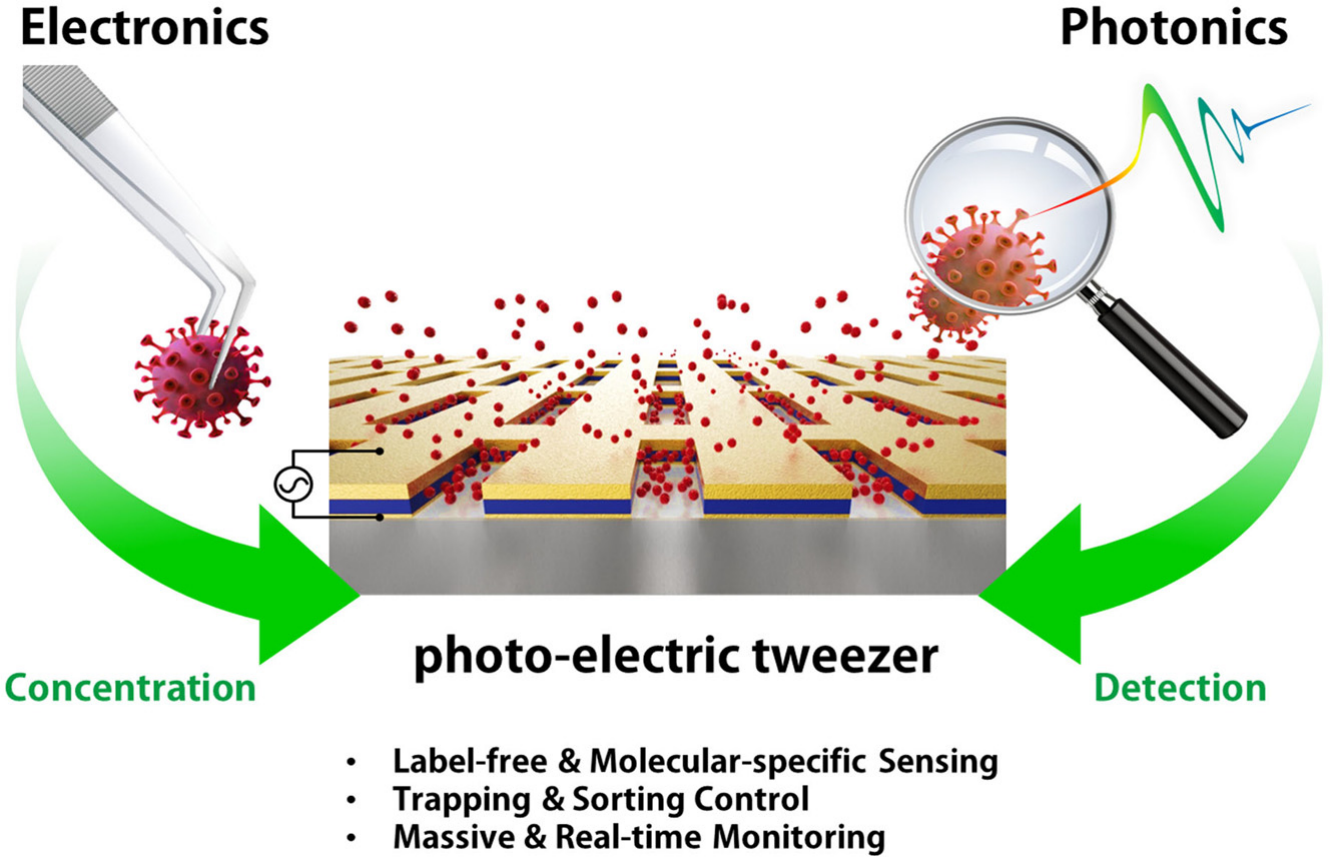
Conceptual illustration of photo-electric tweezers for biochemical substances detection on the metasurface by physical matching of particle capturing sites to the optical sensing hotspots.

Very recently, integrated THz nanoslot metasurface with electric tweezers provided solutions for real-time underwater detection of sub-50 nm bioparticles using broadband wavelength. Not only demonstration of extremely enhanced terahertz reflection, but also concentration of suspended target analytes into the optical hotspots open up the high throughput analysis as well as label-free capturing of analytes in a molecular selective manner. With a help of long-range ACEO collection, remote particles move to the vicinity of nanoslot and they are captured and concentrated at the optical hotpots leading to underwater THz molecular detection in ultra-low molecular populations (Figure 12). This efficient process for biomolecular detection thanks to the vertically-aligned electrode, called Sandwich metasurface. The Sandwich metasurface plays multi-functional roles as an ‘electric tweezer’ for (i) continuous nano/bioparticle providers by the generation of ACEO flows, (ii) a ‘particle collector’ via generation of enhanced dielectrophoretic forces, and (iii) a ‘particle detector’ for monitoring real-time growth of target molecules even within the nano-volumes. This unique platform adopts requisite techniques from the various conventional particle manipulation techniques for realization of the efficient underwater-working photonic sensor; This can be (i) a continuous particle delivery from the ‘microfluidic technique’, (ii) long-range order of particle conveying from the ‘ACEO technique’, and (iii) massive and high-throughput particle gathering from ‘DEP technique’. As the vertically-aligned Sandwich metasurface chip overcame the underwater THz detection of nano-bio objects and allows real-time monitoring of nano-volumetric particle dynamics, this photo-electric tweezer will create significant opportunities to realize advanced metasurface-sensitive analytical devices owing to its facile applicability to a variety of photonic nanogeometry designed for specific optical spectrum. Of course, this will lead to knowledgeable discovery at the multidisciplinary scientific area, contributing to scientific advances as well as bioscience industries.
